# Targeted proteomics reveals promising biomarkers of disease activity and organ involvement in antineutrophil cytoplasmic antibody-associated vasculitis

**DOI:** 10.1186/s13075-017-1429-3

**Published:** 2017-09-29

**Authors:** Jun Ishizaki, Ayako Takemori, Koichiro Suemori, Takuya Matsumoto, Yoko Akita, Ken-ei Sada, Yukio Yuzawa, Koichi Amano, Yoshinari Takasaki, Masayoshi Harigai, Yoshihiro Arimura, Hirofumi Makino, Masaki Yasukawa, Nobuaki Takemori, Hitoshi Hasegawa, for the Research Committee of Intractable Vasculitis Syndrome and the Research Committee of Intractable Renal Disease of the Ministry of Health, Labour and Welfare of Japan, Yohko Murakawa, Eri Muso, Atsushi Komatsuda, Satoshi Ito, Takao Fujii, Atsushi Kawakami, Izaya Nakaya, Takao Saito, Takafumi Ito, Nobuhito Hirawa, Masahiro Yamamura, Masaaki Nakano, Kosaku Nitta, Makoto Ogura, Taio Naniwa, Shoichi Ozaki, Junichi Hirahashi, Noriyoshi Ogawa, Tatsuo Hosoya, Takashi Wada, Satoshi Horikoshi, Yasushi Kawaguchi, Taichi Hayashi, Masaharu Yoshida, Tsuyoshi Watanabe, Daijo Inaguma, Kazuhiko Tsuruya, Noriyuki Homma, Tsutomu Takeuchi, Naoki Nakagawa, Shinichi Takeda, Ritsuko Katabuchi, Masayuki Iwano, Tatsuya Atsumi, Shoichi Fujimoto, Shogo Banno, Takahiko Sugihara, Masaki Kobayashi, Kunihiro Yamagata, Sakae Homma, Hiroaki Dobashi, Naotake Tsuboi, Akihiro Ishizu, Hitoshi Sugiyama

**Affiliations:** 1 Department of Hematology, Clinical Immunology, and Infectious Diseases, Ehime University Graduate School of Medicine, Toon, Ehime 791-0295 Japan; 2 Division of Proteomics Research, Proteo-Science Center, Ehime University, Toon, Ehime 791-0295 Japan; 3 Department of Nephrology, Rheumatology, Endocrinology and Metabolism, Okayama University Graduate School of Medicine, Dentistry and Pharmaceutical Sciences, Okayama, Japan; 4 Department of Nephrology, Fujita Health University School of Medicine, Aichi, Japan; 5 Department of Rheumatology and Clinical Immunology, Saitama Medical Center, Saitama Medical University, Saitama, Japan; 6 Department of Rheumatology, Juntendo University Koshigaya Hospital, Saitama, Japan; 7 Division of Epidemiology and Pharmacoepidemiology of Rheumatic Diseases, Institute of Rheumatology, Tokyo Women’s Medical University, Tokyo, Japan; 8 Nephrology and Rheumatology, First Department of Internal Medicine, Kyorin University School of Medicine, Tokyo, Japan; 9 Okayama University Hospital, Okayama, Japan

**Keywords:** Antineutrophil cytoplasmic antibody-associated vasculitis, Biomarkers, Targeted proteomics, Microscopic polyangiitis, Granulomatosis with polyangiitis, Eosinophilic granulomatosis with polyangiitis

## Abstract

**Background:**

Targeted proteomics, which involves quantitative analysis of targeted proteins using selected reaction monitoring (SRM) mass spectrometry, has emerged as a new methodology for discovery of clinical biomarkers. In this study, we used targeted serum proteomics to identify circulating biomarkers for prediction of disease activity and organ involvement in antineutrophil cytoplasmic antibody (ANCA)-associated vasculitis (AAV).

**Methods:**

A large-scale SRM assay targeting 135 biomarker candidates was established using a triple-quadrupole mass spectrometer coupled with nanoflow liquid chromatography. Target proteins in serum samples from patients in the active and remission (6 months after treatment) stages were quantified using the established assays. Identified marker candidates were further validated by enzyme-linked immunosorbent assay using serum samples (*n* = 169) collected in a large-cohort Japanese study (the RemIT-JAV-RPGN study).

**Results:**

Our proteomic analysis identified the following proteins as biomarkers for discriminating patients with highly active AAV from those in remission or healthy control subjects: tenascin C (TNC), C-reactive protein (CRP), tissue inhibitor of metalloproteinase 1 (TIMP1), leucine-rich alpha-2-glycoprotein 1, S100A8/A9, CD93, matrix metalloproteinase 9, and transketolase (TKT). Of these, TIMP1 was the best-performing marker of disease activity, allowing distinction between mildly active AAV and remission. Moreover, in contrast to CRP, serum levels of TIMP1 in patients with active AAV were significantly higher than those in patients with infectious diseases. The serum levels of TKT and CD93 were higher in patients with renal involvement than in those without, and they predicted kidney outcome. The level of circulating TNC was elevated significantly in patients with lung infiltration. AAV severity was associated with markers reflecting organ involvement (TKT, CD93, and TNC) rather than inflammation. The eight markers and myeloperoxidase (MPO)-ANCA were clustered into three groups: MPO-ANCA, renal involvement (TKT and CD93), and inflammation (the other six markers).

**Conclusions:**

We have identified promising biomarkers of disease activity, disease severity, and organ involvement in AAV with a targeted proteomics approach using serum samples obtained from a large-cohort Japanese study. Especially, our analysis demonstrated the effectiveness of TIMP1 as a marker of AAV activity. In addition, we identified TKT and CD93 as novel markers for evaluation of renal involvement and kidney outcome in AAV.

**Electronic supplementary material:**

The online version of this article (doi:10.1186/s13075-017-1429-3) contains supplementary material, which is available to authorized users.

## Background

The antineutrophil cytoplasmic antibody (ANCA)-associated vasculitides (AAV) comprise three distinct diseases: granulomatosis with polyangiitis (GPA), microscopic polyangiitis (MPA), and eosinophilic granulomatosis with polyangiitis (EGPA) [1]. AAV are characterized by pauci-immune necrotizing inflammation of small to medium-sized vessels and affect multiple organs. ANCA have been shown to play a potential role in the pathogenesis of vasculitis [2]. However, use of ANCA for monitoring disease activity is insufficiently sensitive or specific [3–7]. Traditional acute-phase indicators, including C-reactive protein (CRP), also lack the sensitivity and specificity for monitoring of AAV disease activity. Therefore, additional markers are needed as a guide for management and for distinguishing active disease from remission. Moreover, biomarkers that reflect organ damage are also necessary because AAV affects multiple organs. Several circulating biomarkers allowing comparison of active disease with remission in AAV have been reported in large-cohort studies and systematic literature searches [8–14]. However, these biomarkers are not yet recognized as clinically useful for monitoring of disease activity or predicting prognosis.

Targeted proteomics involves large-scale protein quantification using selected reaction monitoring (SRM; also known as multiple reaction monitoring), and its application for clinical biomarker discovery has been explored in recent years [15–24]. SRM is a quantitative mass spectrometry (MS) technique for selective detection of targeted molecules in a complex analytical sample, and it has become a broadly acceptable approach for protein quantification without the use of antibodies [15, 17, 18]. The multiple target selectivity of SRM is particularly useful for parallel monitoring of different marker proteins, enabling highly sensitive quantitation of proteins in a crude serum sample containing the proteins of interest at subfemtomolar levels. The targeted proteomics approach consists of three steps: (1) selection of novel biomarker candidates based on experimental and/or bioinformatics information, (2) quantitative evaluation of candidate biomarkers using SRM, and (3) verification of selected candidates using an antibody-based quantitative technology such as an enzyme-linked immunosorbent assay (ELISA). This facilitates high-throughput identification of reliable biomarker candidates from limited-availability biological samples.

To analyze the characteristics of Japanese patients with AAV, the Research Committee for Intractable Vasculitis Syndrome and the Research Committee for Intractable Renal Disease of the Ministry of Health, Labour and Welfare of Japan collaboratively implemented a nationwide prospective cohort study of remission induction therapy in Japanese patients with antineutrophil cytoplasmic antibody-associated vasculitis and rapidly progressive glomerulonephritis (RemIT-JAV-RPGN) [25]. In contrast to studies [8, 9, 13] related to the RAVE trial, which have indicated that GPA with positive proteinase-3 (PR3) is predominant in patients with AAV from the United States and The Netherlands, Japanese patients with AAV appear to have a higher incidence of MPA than of GPA, and they show a predominance of myeloperoxidase (MPO)-ANCA. In the present study, we employed targeted proteomics to identify novel circulating protein biomarkers of disease activity and severity, as well as organ damage, in AAV using serum samples obtained from a large-cohort Japanese study (RemIT-JAV-RPGN).

## Methods

### Healthy donors and patients

Serum samples from patients with AAV were obtained at our hospital (*n* = 17) and from a cohort study (RemIT-JAV-RPGN) (*n* = 152) [25]. All cases of AAV fulfilled the criteria for primary systemic vasculitis proposed by the European Medicines Agency (EMEA) algorithm [26]. From among 321 patients who had been enrolled initially in the RemIT-JAV-RPGN study, serum samples for use in the present study were collected from 152 patients with active disease (before treatment) and 64 patients at 6 months after the start of treatment. On the basis of the EMEA algorithm, 169 of these patients were diagnosed as follows: 105 with MPA, 36 with GPA, 25 with EGPA, and 3 with unclassifiable disease. Paired serum samples before and 6 months after the start of treatment were obtained from 79 patients: 42 with MPA, 20 with GPA, 14 with EGPA, and 3 with unclassifiable disease.

Serum samples were also obtained from 30 healthy donors, 30 patients with active rheumatoid arthritis (RA), 21 patients with active systemic lupus erythematosus (SLE), and 25 patients with bacterial infectious diseases treated at our hospital. RA was diagnosed on the basis of the 2010 American College of Rheumatology (ACR)/European League Against Rheumatism (EULAR) criteria for RA [27]. The criterion for the active phase of RA in this study was considered to be a Simplified Disease Activity Index ≥ 11 [28]. Patients with SLE fulfilled the ACR revised criteria for SLE [29]. The active phase of SLE was defined as a Systemic Lupus Erythematosus Disease Activity Index score ≥ 4, based on clinical findings in the 2 weeks prior to sample collection [30]. The 25 patients with infectious diseases included 15 with bacterial pneumonia, 4 with urinary infection, 5 with acute cholecystitis, and 1 with enterocolitis. Information on the characteristics of the healthy donors and patients is given (Table 1, Additional file 1: Table S1). Serum samples were frozen at −80 °C until use.

**Table 1:** Baseline characteristics of the 169 patients with antineutrophil cytoplasmic antibody-associated vasculitis

	All (*n* = 169)	MPA (*n* = 105)	GPA (*n* = 36)	EGPA (*n* = 25)
Male/female, *n*/*n*	65/104	45/60	14/22	6/19
Age, years	71 (61–78)	73 (66–78)	69 (60–79)	60 (46–70)
MPO-ANCA-positive	137 (81.1%)	103 (98.1%)	22 (61.1%)	9 (36%)
PR3-ANCA-positive	20 (12.2%)	4 (4.0%)	15 (41.7%)	1 (4.2%)
ANCA-negative	18 (10.7%)	1 (1.0%)	1 (2.8%)	16 (64%)
WBC, count/μl	9400 (7000–14,300)	8500 (6200–12,300)	9950 (7825–14,050)	19,200 (15,090–27,700)
Serum creatinine, mg/dl	0.92 (0.66–2.43)	1.41 (0.81–3.56)	0.71 (0.61–1.24)	0.59 (0.44–0.76)
eGFR, ml/minute/1.73 m^2^	50 (17–74)	34 (12–61)	65 (41–75)	81 (68–107)
Disease activity and organ involvement				
BVAS score	16 (12–21)	15 (12–19)	19 (12–24)	17 (14–20)
BVAS chest positive	75 (44.4%)	43 (41%)	17 (47.2%)	15 (60%)
BVAS renal positive	120 (71%)	92 (87.6%)	23 (63.9%)	4 (16%)
Disease severity				
EUVAS disease severity, L/ES/Ge/Se, *n*	5/37/96/31	2/27/52/24	3/7/23/3	0/1/20/14
RPGN clinical grading, I/II/III/IV, *n*	40/86/38/5	18/54/28/5	8/21/7/0	14/9/2/0
Five-Factor Score, ≤ 1/2/≥ 3, *n*	67/57/45	18/43/44	29/7/0	20/4/1
Outcomes at 6 months				
Clinical remission (BVAS score = 0)	121/157 (77.1%)	71/95 (74.7%)	28/35 (80%)	19/24 (79.2%)
ESRD	15/157 (9.6%)	14/95 (14.7%)	1/35 (2.9%)	0/24 (0.0%)

*Abbreviations: eGFR* Estimated glomerular filtration rate, *BVAS* Birmingham Vasculitis Activity Score version 3, *EUVAS* European Vasculitis Study Group, *L/ES/Ge/Se* Localized/early systemic/generalized/severe, *RPGN* Rapidly progressive glomerulonephritis, *ESRD* End-stage renal disease, *MPO* Myeloperoxidase, *ANCA* Antineutrophil cytoplasmic antibody, *PR3* Proteinase-3, *WBC* White blood cell count, *EGPA* Eosinophilic granulomatosis with polyangiitis, *GPA* Granulomatosis with polyangiitis, *MPA* Microscopic polyangiitis

Values are median (IQR) or *n* (%)

### Outcome measures

Details of the RemIT-JAV-RPGN study protocol were reported previously [25]. Disease activity was evaluated according to the Birmingham Vasculitis Activity Score version 3 (BVAS) system [31]. Remission was defined as complete absence of disease activity attributable to active vasculitis. Absence of disease activity was determined systematically using a BVAS of 0 on two occasions at least 1 month apart according to the EULAR recommendations [32]. Organ involvement was evaluated in accordance with the BVAS system. Renal damage was defined as a renal BVAS score ≥ 1. End-stage renal disease (ESRD) was defined as dependence on dialysis or an irreversible increase in the serum creatinine level to > 5.6 mg/dl. Lung involvement was defined as a chest BVAS score ≥ 1.

### Disease severity

The disease severity of the patients enrolled in the RemIT-JAV-RPGN study was classified as localized, early systemic, generalized, or severe in accordance with the definition of the European Vasculitis Study Group (EUVAS) [32]. Patients with threatened vital organ function were classified as having generalized disease, and patients with organ failure were classified as having severe disease. The definitions of disease severity have been described in detail previously [25].

The patients were also classified into four groups according to the Japanese rapidly progressive glomerulonephritis (RPGN) clinical grading [33], which considers the following parameters: serum creatinine level (<3 mg/dl = 0 points, 3–6 mg/dl = 1 point, > 6 mg/dl = 2 points, and dialysis-dependent = 3 points), age (≤ 59 years = 0, 60–69 years = 1, and ≥ 70 years = 2), lung involvement (negative = 0 and positive = 2), and serum CRP level (< 2.6 mg/dl = 0, 2.6–10.0 mg/dl = 1, and > 10 mg/dl = 2). Lung involvement was defined as the presence of chest symptoms in BVAS or interstitial lung disease (ILD). In the present study, ILD was diagnosed by site investigators using chest radiography and/or thoracic computed tomography. The point totals for these four parameters were summed, and patients were divided into four groups as follows: grade I, 0–2 points; grade II, 3–5 points; grade III, 6–7 points; and grade IV, 8–9 points.

For calculation of the Five-Factor Score (FFS) 2009 [34], renal insufficiency was defined as a serum creatinine level > 1.7 mg/dl; cardiac insufficiency as the presence of cardiac symptoms in BVAS; gastrointestinal involvement as the presence of abdominal symptoms in BVAS; and ear, nose, and throat (ENT) involvement as the presence of ENT symptoms in BVAS. Age > 65 years, cardiac insufficiency, gastrointestinal involvement, and renal insufficiency were each accorded +1 point, and absence of ENT manifestations was also accorded +1 point. The patients were divided into three groups according to the summed point totals for these five parameters: ≤ 1, 2, or ≥ 3.

### Protein digestion

Protein digestion for SRM analysis was performed as described previously [35]. Fourteen major serum proteins were depleted using a Multiple Affinity Removal Spin Cartridge Human-14 (Agilent Technologies, Santa Clara, CA, USA). For sample preparation prior to liquid chromatography-tandem mass spectrometry (LC-MS/MS), serum proteins (5 μg of total protein) separated on NuPAGE 4–12% gel (Life Technologies, Carlsbad, CA, USA) were digested with sequencing grade trypsin (Promega, Madison, WI, USA) in 100 mM ammonium bicarbonate (pH 8.8) overnight at 37 °C. Digested peptides were desalted using a self-made C18 STop And Go Extraction tip and eluted with 40 μl of 0.1% (vol/vol) trifluoroacetic acid (TFA)/80% (vol/vol) acetonitrile. Eluates were dried by vacuum centrifugation and reconstituted with 10 μl of 0.1% (vol/vol) TFA for MS analysis.

### Mass spectrometry

MS/MS and SRM analyses were carried out on a QTRAP 5500 mass spectrometer equipped with a nanoelectrospray ionization source (SCIEX, Framingham, MA, USA) as described previously [21, 24]. Chromatographic separation of the digested peptides was performed using an Eksigent NanoLC system (SCIEX). The mobile phases consisted of 0.1% (vol/vol) formic acid in H_2_O as solvent A and 0.1% (vol/vol) formic acid/80% (vol/vol) acetonitrile as solvent B. For MS/MS analysis, peptide samples were injected onto a 200-μm inner diameter (i.d.) × 0.5-mm cHiPLC trap column (SCIEX). Concentrated peptides were then separated on a 75-μm i.d.  × 15-cm C18 reversed-phase cHiPLC column (SCIEX) at a flow rate of 300 nl/minute using the following gradient schedule: 0–60 minutes, 2–18% B; 60–95 minutes, 18–40% B; 95–100 minutes, 40–90% B; holding at 90% B for 5 minutes, and re-equilibration at 2% B for 15 minutes. MS/MS spectra were searched against the UniProt human proteome database using ProteinPilot software version 4.1 (SCIEX) with the following parameters: cysteine alkylation, acrylamide; digestion, trypsin; processing parameters, biological modification; and search effort, through ID.

LC-SRM assays were developed using [^13^C_6_,^15^N_2_]lysine-labeled or [^13^C_6_,^15^N_4_]arginine-labeled standard peptides (Sigma-Aldrich, St. Louis, MO, USA) as described previously [21, 24]. For SRM analysis using the LC-MS system, targeted peptides were separated at a flow rate of 300 nl/minute employing the following gradient schedule: 0–60 minutes, 2–18% B; 60–95 minutes, 18–40% B; 95–100 minutes, 40–90% B; holding at 90% B for 5 minutes, and re-equilibration at 2% B for 15 minutes.

Quantitative analysis of the obtained SRM data was performed using Skyline software [36]. Quantification of relative protein abundance across different serum samples is based on the peak area ratios of the light (endogenous) and heavy (^13^C/^15^N-labeled internal standard) forms of each peptide.

### ELISA

Analysis of samples was performed using commercially available ELISA kits in accordance with the manufacturer’s instructions. We measured heterodimer S100A8/A9 because S100A8 and S100A9 form a heterodimer. The following ELISA kits were used: CRP, CD93, matrix metalloproteinase 9 (MMP9), S100A8/A9, and tissue inhibitor of metalloproteinase 1 (TIMP1) (R&D Systems, Minneapolis, MN, USA); leucine-rich alpha-2-glycoprotein 1 (LRG1) and tenascin C (TNC) (Immuno-Biological Laboratories, Gunma, Japan); transketolase (TKT) (LifeSpan BioSciences, Seattle, WA, USA); and MPO-ANCA (MBL, Nagoya, Japan).

### Statistical analysis

Values are expressed as medians and IQRs or as numbers and percentages. Continuous nonparametric variables were compared using the Mann-Whitney *U* test. Distinction of active AAV from remission in the same patients with AAV was compared using the Wilcoxon signed-rank test. Analysis of covariance was employed for comparison analysis when adjustment was necessary for age, sex, and four distinct AAV groups (MPA, GPA, EGPA, and unclassifiable disease) to calculate the adjusted means for each biomarker level. When using analysis of covariance, each biomarker level was logarithmically transformed to attain a normal distribution and then compared using Student’s *t* test and Dunnett’s test. For easier reading, biomarker levels presented in the tables and figures were not transformed. ROC curves were constructed for each marker using logistic regression to further assess the ability of the biomarkers and to define the optimal cutoff point. The AUC was calculated, and positive likelihood ratios (LRs) (sensitivity/[1 − specificity]) were determined at the optimal cutoff points. Correlations between paired data were analyzed using Spearman’s rank correlation. Differences at *p* < 0.05 were considered to be statistically significant. When comparing 44 biomarker candidates in protocol 1 or 15 in protocol 2 identified by SRM assay between active AAV and remission, statistical significance was determined by < 0.05/44, or < 0.05/15 by Bonferroni correction to adjust for multiple testing. Statistical analyses were performed using JMP version 9 software (SAS Institute, Cary, NC, USA).

## Results

### Candidate screening and assay development

**Fig. 1:**
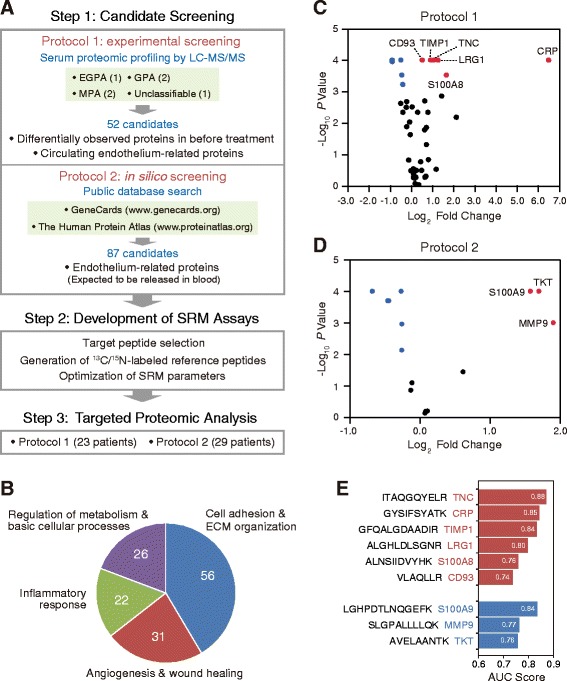
Identification of candidate biomarkers of disease activity in antineutrophil cytoplasmic antibody-associated vasculitis (AAV) using the selected reaction monitoring (SRM) assay. **a** Workflow for the development of SRM assays targeting marker candidates of disease activity in AAV. **b** Functional classification of marker candidates. **c** Comparison of 44 candidate markers discriminating between highly active AAV (before treatment) and remission (at 6 months after the start of treatment) using the SRM assay in protocol 1. Paired serum samples prepared from 23 patients (9 microscopic polyangiitis [MPA], 7 granulomatosis with polyangiitis [GPA], 5 eosinophilic granulomatosis with polyangiitis [EGPA], and 2 unclassifiable) were used in protocol 1. **d** Comparison of 15 candidate markers discriminating between highly active AAV and remission using the SRM assay in protocol 2. Paired serum samples prepared from 29 patients (10 MPA, 9 GPA, and 10 EGPA) were used in protocol 2. Volcano plots are used to look at fold changes and statistical significance simultaneously. Dot plots show − log_10_ (*p* values) on the ordinate and log_2_ (fold change values) on the abscissa for all activity marker candidates. The upper corners of the plot (*red* and *blue dots*) represent proteins that show both statistical significance and large fold changes. *Red* and *blue dots* indicate the proteins that were downregulated and upregulated after treatment, respectively. *p* Values were determined by Wilcoxon signed-rank test, and statistical significance was determined by < 0.05/44 or < 0.05/15 by Bonferroni correction. **e** Proteins downregulated after treatment with AUC scores > 0.7 in protocols 1 and 2. The upper six proteins were identified in protocol 1 and the lower three proteins in protocol 2. *CRP* C-reactive protein, *ECM* Extracellular matrix, *LRG1* Leucine-rich alpha-2-glycoprotein 1, *MMP9* Matrix metalloproteinase 9, *TIMP1* Tissue inhibitor of metalloproteinase 1, *TKT* Transketolase, *TNC* Tenascin C

The workflow for the development of SRM assays targeting marker candidates of disease activity in AAV is shown in Fig. 1a. Initially, we selected the targeted candidates by an experiment-based approach (protocol 1) in which we conducted MS-based serum proteomic profiling of patients with AAV before and after treatment. Paired serum samples before (active) and at 6 months (remission) after treatment were obtained from six patients (one EGPA, two MPA, two GPA, and one unclassifiable). After depletion of high-abundance proteins with an immunoaffinity column, the remaining proteins were subjected to LC-MS/MS analysis (Additional file 1: Figure S1a). From among the 267 proteins identified (Additional file 2: Table S2), we selected 52 proteins as candidate markers on the basis of the following criteria: (1) those specifically observed before treatment, (2) those that showed significant differences in the number of identified peptides (> 1.5-fold) before and after treatment, or (3) those that were endothelium-related and present in serum (Additional file 1: Figure S1b).

To maximize the chance of discovering marker proteins in this study, we further selected 87 endothelium-related proteins, which, based on information in publicly available databases, were expected to be present in blood [37, 38] (Fig. 1a, protocol 2). In total, 135 proteins were selected for development of SRM assays (Additional file 1: Table S3). On the basis of their biological functions, we classified the identified proteins into four groups (Fig. 1b) involved in the following: (1) cell adhesion and extracellular matrix organization (56 proteins), (2) angiogenesis and wound healing (31 proteins), (3) inflammatory responses (22 proteins), and (4) regulation of metabolism and basic cellular processes (26 proteins).

Development of a reliable SRM assay generally requires predetermination of target peptides that are suitable for SRM quantification. For all the proteins in protocol 1, we selected the target peptides from the peptide dataset observed in our MS/MS analysis. Owing to the lack of available MS/MS information for the proteins in protocol 2, we referenced the MS-based proteomic information in public databases (PeptideAtlas [39] and Plasma Proteome Database [40]), and we selected target peptides for the assay construction. All of the peptides selected for assay development are listed in Additional file 1: Table S3. To establish LC-SRM assays targeting all of the selected peptides, we generated ^13^C/^15^N-labeled synthetic peptide analogues for use as standard reference material in SRM analysis. MS/MS data obtained from synthesized reference peptides were used to generate a spectral library for SRM assay development. On the basis of the established library, the fragment ions for each of the precursor ions were selected for the final assay (Additional file 1: Figure S2).

Although SRM has a high capability to detect targeted proteins in biological samples, it is always necessary to reduce the complexity of the sample for analysis of the minor protein components. In this study, we conducted immunoaffinity depletion of the 14 most abundant proteins to enhance the SRM detectability of proteins with lower abundance. On one hand, the sample pretreatment in our experimental workflow enabled us to detect the targeted serum proteins at a low ng/ml level in a 10-μl sample. On the other hand, SRM quantification of low-abundance proteins at the picograms per milliliter level (e.g., cytokines and chemokines in human serum) still challenges current MS capability. In addition, we excluded complement components, which show physiologically relevant interactions with immune-depleted proteins during sample pretreatment, from consideration as targeted candidates for SRM assay.

### Targeted proteomics of selected candidates

Using the established SRM assay, we performed parallel monitoring of target peptides and obtained LC-SRM chromatographs of each peptide from paired serum samples (before and at 6 months after treatment of the same patients) that were prepared from 23 patients (9 MPA, GPA 7, 5 EGPA, and 2 unclassifiable) in protocol 1 and from 29 patients (10 MPA, 9 GPA, and 10 EGPA) in protocol 2. Of the 135 marker candidates, 74 proteins were successfully quantified in this study: 44 in protocol 1 and 30 in protocol 2 (Additional file 1: Table S4). However, in protocol 2, some proteins that were quantified in only a small number of samples were impossible to analyze statistically. Therefore, to select marker candidates using statistical analysis, of the 30 marker candidates, 15 proteins that were quantified in paired serum samples from more than 20 patients were further selected. The 11 proteins in protocol 1 and 9 proteins in protocol 2 showed a significant change before and at 6 months after treatment (Bonferroni correction; *p* < 0.05/44 in protocol 1 and *p* < 0.05/15 in protocol 2) (Fig. 1c, d; Additional file 1: Table S5). We identified downregulated proteins after treatment with AUC > 0.7 as candidate biomarkers of disease activity. Of these proteins, the following nine were identified as candidate biomarkers of disease activity in the SRM assay: TNC, CRP, TIMP1, LRG1, S100A8, and CD93 in protocol 1; and S100A9, MMP9, and TKT in protocol 2 (Fig. 1e).

### Serum levels of candidate biomarkers in active AAV, remission, and healthy control subjects

**Fig. 2:**
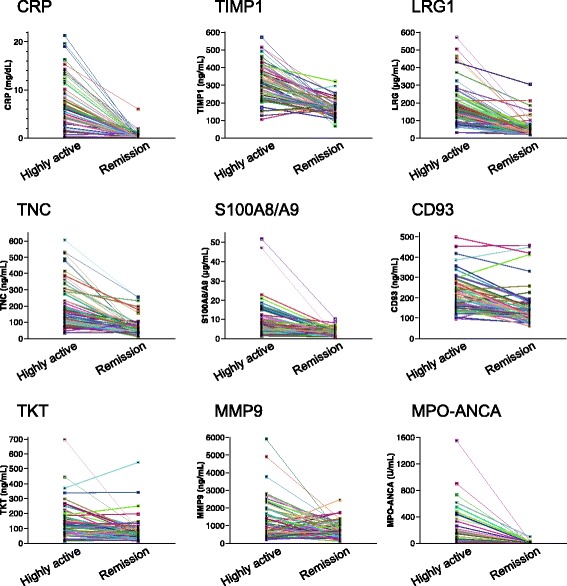
Marker levels in highly active antineutrophil cytoplasmic antibody-associated vasculitis before treatment and remission at 6 months after treatment. The enzyme-linked immunosorbent assay titers of markers were measured in paired serum samples (before and at 6 months after the start of treatment) from the 62 patients with antineutrophil cytoplasmic antibody-associated vasculitis (33 microscopic polyangiitis [MPA], 15 granulomatosis with polyangiitis [GPA], 11 eosinophilic granulomatosis with polyangiitis [EGPA], and 3 unclassifiable). Each line connects the data obtained on one patient. *Myeloperoxidase (MPO)-ANCA* refers to only MPO-ANCA-positive patients (*n* = 48). *CRP* C-reactive protein, *LRG1* Leucine-rich alpha-2-glycoprotein 1, *MMP9* Matrix metalloproteinase 9, *TIMP1* Tissue inhibitor of metalloproteinase 1, *TKT* Transketolase, *TNC* Tenascin C

**Fig. 3:**
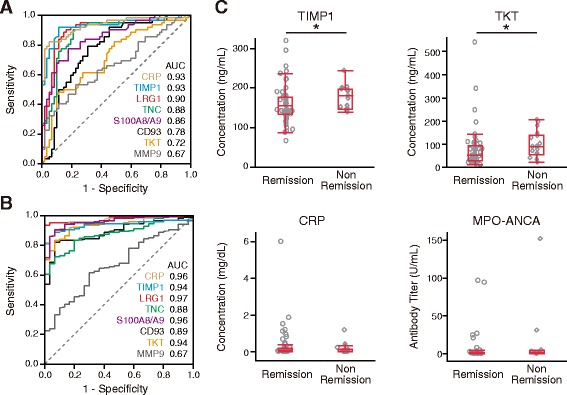
**a** ROC curves and AUC scores showing ability of markers to distinguish highly active antineutrophil cytoplasmic antibody-associated vasculitis (AAV) from remission. The enzyme-linked immunosorbent assay (ELISA) titers of markers were measured in paired serum samples (before and at 6 months after the start of treatment) from the 62 patients with AAV. The 62 patients with highly active AAV had total Birmingham Vasculitis Activity Score version 3 (BVAS) score of 15 (range 12–21). **b** ROC curves and AUC scores showing ability of markers to distinguish highly active AAV from healthy control subjects. The ELISA titers of markers were compared between highly active AAV (*n* = 169, before treatment) and healthy control subjects (*n* = 30). The 169 patients before treatment had total BVAS score of 16 (range 12-21). c Comparison of marker levels between the remission group (*n* = 62) and the nonremission group (*n* = 17) at 6 months after the start of treatment. The 17 nonremission patients (9 microscopic polyangiitis [MPA], 5 granulomatosis with polyangiitis [GPA], and 3 eosinophilic granulomatosis with polyangiitis [EGPA]) had very mildly active AAV (total BVAS 5, range 3–6). Each *dot* represents one patient. Box plots show median and IQR. Whiskers indicate most extreme points within 1.5 times the IQR of the box. *p* Values were determined by analysis of covariance adjusted for age, sex, and four distinct AAV groups (MPA, GPA, EGPA, and unclassifiable disease). *p < 0.05 (significant difference). *CRP* C-reactive protein, *LRG1* Leucine-rich alpha-2-glycoprotein 1, *MMP9* Matrix metalloproteinase 9, *TIMP1* Tissue inhibitor of metalloproteinase 1, *TKT* Transketolase, *TNC* Tenascin C

To validate the candidate biomarkers selected by SRM, we analyzed the levels of such candidates in paired AAV serum samples before (active) and at 6 months after treatment (remission), as well as in samples from healthy control subjects, using commercial ELISA kits. In the preliminary experiment, we confirmed that the ELISA titers of markers measured in this study corresponded well with the light/heavy ratio of SRM (Additional file 1: Figure S3). The results of marker measurements in samples from active AAV and remission are shown in Figs. 2 and 3a and Table 2. Sixty-two patients who were in remission at 6 months comprised 33 with MPA, 15 with GPA, 11 with EGPA, and 3 with unclassifiable disease. These patients had highly active AAV before treatment (total BVAS 15, range 12–21). Serum levels of CRP, TIMP1, LRG1, TNC, S100A8/A9, CD93, TKT, and MMP9 were all significantly higher in the active stage than in remission in these 62 patients. In the 48 MPO-ANCA-positive patients, the MPO-ANCA level was also higher in active AAV than in remission. The eight markers except for MMP9 had AUC > 0.7 for differentiation between highly active AAV and remission. Of these, four—CRP, TIMP1, LRG1, and MPO-ANCA—were the best-performing markers with AUC ≥ 0.9 and positive LR 6.11–17.0 for distinguishing highly active AAV from remission.

**Table 2:** Marker levels in highly active antineutrophil cytoplasmic antibody-associated vasculitis before treatment and remission at 6 months

Marker	Highly active (*n* = 62)	Remission (*n* = 62)	*p* Value	AUC^a^	COP^b^	Sensitivity	Specificity	LR + ^c^
CRP, mg/dl	6.34 (2.14–11.5)	0.06 (0.01–0.16)	< 0.0001^d^	0.93	1.22	82	95	17.0
TIMP1, ng/ml	329 (250–405)	156 (134–175)	< 0.0001^d^	0.93	205	92	89	8.14
LRG1, μg/ml	151 (110–231)	40.1 (29.8–62.0)	< 0.0001^d^	0.9	71.9	89	85	6.11
TNC, ng/ml	144 (97.7–272)	47.0 (37.0–68.3)	< 0.0001^d^	0.88	72.7	90	79	4.31
S100A8/A9, μg/ml	6.4 (4.4–10.3)	2.2 (1.2–3.8)	< 0.0001^d^	0.86	4.1	77	82	4.36
CD93, ng/ml	192 (151–252)	124 (103–164)	< 0.0001^d^	0.78	146	79	69	2.58
TKT, ng/ml	104 (61.8–162)	52.0 (28.9–91.5)	< 0.0001^d^	0.72	115	47	90	4.83
MMP9, ng/ml	909 (447–1623)	566 (330–839)	0.0007^d^	0.67	897	53	77	2.36
MPO-ANCA,^e^ U/ml	63.5 (27.4–223)	0.8 (0.5–2.2)	< 0.0001^d^	0.96	5.1	98	88	7.83

*Abbreviations: ANCA* Antineutrophil cytoplasmic antibody, *COP* Cutoff point, *CRP* C-reactive protein, *LR* Likelihood ratio, *LRG1* Leucine-rich alpha-2-glycoprotein 1, *MMP9* Matrix metalloproteinase 9, *MPO* Myeloperoxidase, *TIMP1* Tissue inhibitor of metalloproteinase 1, *TKT* Transketolase, *TNC* Tenascin C

Values are median (IQR)

^a^An AUC of 1 indicates perfect discrimination between groups; an AUC of 0.5 indicates no discrimination

^b^The maximum sum of sensitivity and specificity

^c^Positive likelihood ratio at the COP, which equals sensitivity/(1 − specificity)

^d^ *p* < 0.05 by Wilcoxon signed-rank test

^e^Only MPO-ANCA-positive patients (*n* = 48)

A comparison of these markers between patients with active AAV and healthy control subjects is shown in Fig. 3b and Table 3. Seven markers, with the exception of MMP9, also had AUC > 0.8 for distinguishing between active AAV and healthy control subjects. Of these, five—CRP, TIMP1, LRG1, S100A8/A9, and TKT—were the best-performing markers with AUC > 0.9 and positive LR 12.5–25.9 for distinguishing patients with active AAV from control subjects.

**Table 3:** Marker levels in patients with highly active antineutrophil cytoplasmic antibody-associated vasculitis before treatment and healthy control subjects

Marker	Active AAV (*n* = 169)	Healthy control subjects (*n* = 30)	*p* Value	AUC^a^	COP^b^	Sensitivity	Specificity	LR + ^c^
CRP, mg/dl	3.79 (0.9–8.24)	0.01 (0.005–0.03)	< 0.0001^d^	0.96	0.16	86	97	25.9
TIMP1, ng/ml	333 (226–426)	132 (115–148)	< 0.0001^d^	0.94	183	86	97	25.9
LRG1, μg/ml	86.1 (53.7–134)	16.3 (13.6–20.7)	< 0.0001^d^	0.97	28.8	93	100	–
TNC, ng/ml	145 (84.3–265)	48.0 (35.4–64.3)	< 0.0001^d^	0.88	93.1	72	93	10.8
S100A8/A9, μg/ml	5.8 (3.5–8.9)	1.3 (1.1–1.8)	< 0.0001^d^	0.96	2.2	91	93	13.6
CD93, ng/ml	226 (167–300)	125 (103–139)	0.0005^d^	0.89	149	82	93	12.3
TKT, ng/ml	124 (69.7–183)	33.1 (15.1–42.6)	< 0.0001^d^	0.94	58.1	83	93	12.5
MMP9, ng/ml	791 (429–1382)	517 (294–838)	0.0158^d^	0.67	642	62	70	2.05

*Abbreviations: ANCA* Antineutrophil cytoplasmic antibody, *COP* Cutoff point, *CRP* C-reactive protein, *LR* Likelihood ratio, *LRG1* Leucine-rich alpha-2-glycoprotein 1, *MMP9* Matrix metalloproteinase 9, *MPO* Myeloperoxidase, *TIMP1* Tissue inhibitor of metalloproteinase 1, *TKT* Transketolase, *TNC* Tenascin C

Values are median (IQR)

^a^An AUC of 1 indicates perfect discrimination between groups; an AUC of 0.5 indicates no discrimination

^b^COP is the maximum sum of sensitivity and specificity

^c^LR+ at the COP, which equals sensitivity/(1 − specificity)

^d^ *p* < 0.05 by analysis of covariance adjusted for age and sex

Next, we examined whether these markers were able to distinguish between nonremission (mildly active AAV) and remission at 6 months after treatment. Seventeen patients, nine with MPA, five with GPA, and three with EGPA, were in nonremission and had very mildly active AAV (total BVAS 5, range 3–6). As shown in Fig. 3c and Additional file 1: Table S6, TIMP1 and TKT were significantly higher at 6 months in the 17 nonremission patients than in the 62 remission patients. The TIMP1 cutoff point for remission was < 144 ng/ml with a sensitivity of 47% and a specificity of 94%, whereas that for TKT was < 52 ng/ml with a sensitivity of 52% and a specificity of 82%. There were no significant differences in the levels of TIMP1 and TKT between young and old healthy donors (Additional file 1: Table S7). In contrast, the serum levels of seven other markers—CRP, LRG1, TNC, S100A8/A9, CD93, MMP9, and MPO-ANCA—showed no significant difference between nonremission and remission at 6 months. Taken together, these findings indicated that TIMP1 was the best-performing marker for distinguishing mildly or highly active AAV from remission or from healthy control subjects.

### Correlation of marker levels with AAV disease activity

We analyzed the correlation of each marker with the total BVAS score at the active stage (before treatment). The levels of seven markers—TKT, TIMP1, S100A8/A9, CD93, TNC, CRP, and LRG1—were correlated with total BVAS score, but the correlation was modest, with coefficients of 0.18–0.35 (Additional file 1: Figure S4). Among these markers, the strongest correlation with total BVAS score was seen for TKT (coefficient 0.35). In contrast, MPO-ANCA and MMP9 showed no correlation with the total BVAS score.

### Association of marker levels with organ involvement in AAV

**Fig. 4:**
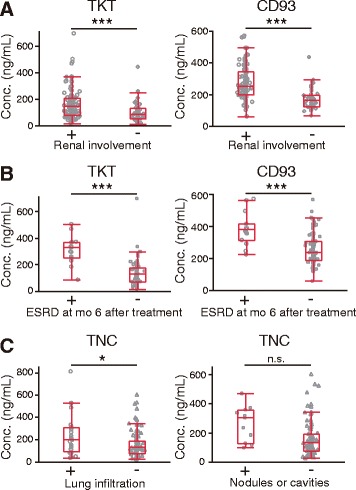
Association of marker levels with renal and lung involvement of antineutrophil cytoplasmic antibody-associated vasculitis (AAV). **a** Comparison of transketolase (TKT) and CD93 levels between patients with AAV with (*n* = 120) and without (*n* = 49) renal involvement before treatment. **b** Comparison of TKT and CD93 levels at baseline between patients with AAV who did (*n* = 15) and did not (*n* = 97) develop end-stage renal disease (ESRD) at 6 months after the start of treatment. **c** Comparison of tenascin C (TNC) levels between patients with AAV with and without lung involvement before treatment. The numbers of patients with only lung infiltration, only nodules or cavities, and nonlung involvement were 25, 11, and 95, respectively. Statistical analysis is described in the legend of Fig. 3. *p < 0.05; ***p < 0.001. *n.s.* Not significant

We then examined which markers are associated with renal and lung involvement, which are often observed in AAV and affect prognosis. As shown in Fig. 4a and Additional file 1: Table S8, the serum levels of TKT and CD93 at baseline were higher in patients with active renal disease than in those without. Moreover, of the 120 patients with renal involvement, the levels of TKT and CD93 at baseline were also higher in patients who developed ESRD after 6 months than in those who did not develop ESRD (Fig. 4b). The cutoff point of TKT for development of ESRD was > 229 ng/ml with a sensitivity of 87%, a specificity of 93%, and an AUC of 0.91, whereas that for CD93 was > 356 ng/ml with a sensitivity of 73%, a specificity of 86%, and an AUC of 0.82. Because the levels of these markers could be affected by active renal disease and/or decreased glomerular filtration rate (GFR), the effect of GFR on the levels of these markers among patients in remission at 6 months was measured. GFR affected the levels of both TKT (7.4 ng/ml increase per 10 ml/minute/1.73 m^2^ decrease in estimated glomerular filtration rate [eGFR]) and CD93 (13.5 ng/ml increase per 10 ml/minute/1.73 m^2^ decrease in eGFR) among patients in remission. Therefore, GFR appeared to be a modest contributor to the serum levels of TKT and CD93. There were no significant differences of the levels of CD93 and TKT between young and old healthy donors (Additional file 1: Table S7). Markers other than TKT and CD93 showed no significant difference between patients with and without renal disease. These findings suggested that TKT and CD93 might be excellent biomarkers for predicting kidney outcome in AAV.

We then analyzed which markers were higher in patients with AAV with lung involvement than in those without. Only the TNC level was elevated in patients with only lung infiltration, but TNC was not elevated in patients with only nodules or cavities (Fig. 4c, Additional file 1: Table S9). Associations of markers with only wheezing (*n* = 4), only endobronchial involvement (*n* = 0), only massive hemoptysis/alveolar hemorrhage (*n* = 3), and only respiratory failure (*n* = 0) could not be analyzed, owing to the small numbers of patients. Analysis of markers for pleural effusion/pleurisy (*n* = 14) was not appropriate, because the patients included those with other diseases such as heart failure and low protein. These findings suggested that TNC might act as a biomarker reflecting lung infiltration in AAV.

### Comparison of marker levels among MPA, GPA, and EGPA

We compared marker levels among the patients with MPA, GPA, or EGPA before treatment (Additional file 1: Table S10). There were no significant differences in the levels of CRP, TIMP1, and S100A8/A9 among the three types of AAV. The levels of CD93 and TKT were higher in MPA than in GPA or EGPA. This is because a higher proportion of patients with MPA had renal involvement in comparison with those with GPA and EGPA (88%, 64%, and 16%, respectively) (Table 1).

### Association of marker levels with disease severity in AAV

**Fig. 5:**
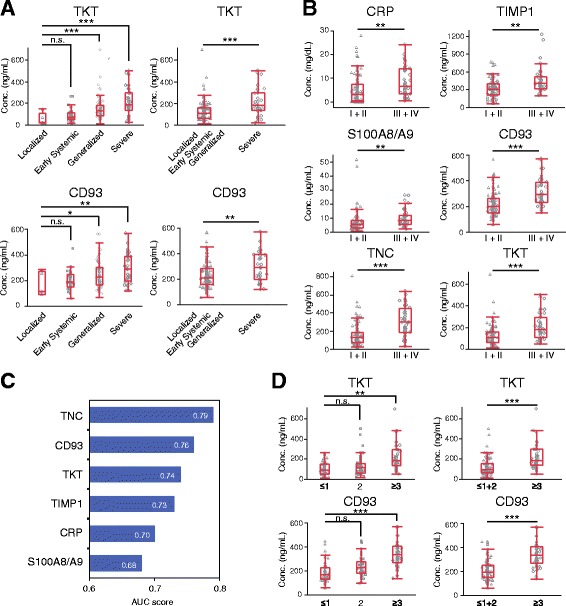
Association of marker levels with disease severity in antineutrophil cytoplasmic antibody-associated vasculitis (AAV). **a** Association of transketolase (TKT) and CD93 levels with European Vasculitis Study Group-defined disease severity. *Left panels*: Comparison of the levels of each marker among the four groups: localized (*n* = 5), early systemic (*n* = 37), generalized (*n* = 96), and severe (*n* = 31) disease. *Right panels*: Comparison of the levels of each marker between patients with severe disease (*n* = 31) and the other three groups (*n* = 138). **b** Association of the level of each marker with rapidly progressive glomerulonephritis (RPGN) clinical grading. Serum levels of each marker were compared between grades I + II (*n* = 126) and grades III + IV (*n* = 43). **c** AUC score for each marker distinguishing between RPGN clinical grades I + II (*n* = 126) and grades III + IV (*n* = 43). **d** Association of TKT and CD93 levels with the Five-Factor Score 2009 (FFS). *Left panels*: Comparison of each marker among the three groups: ≤ l, 2, and ≥ 3 points (≤ 1, *n* = 67; 2, *n* = 57; and ≥ 3, *n* = 45). *Right panels*: Comparison of the level of each marker between the patients with ≤ 1 + 2 points (*n* = 124) and those with ≥ 3 points (*n* = 45). Statistical analysis is described in the Fig. 3 legend. *p < 0.05; **p < 0.01; ***p < 0.001. *n.s.* Not significant, *CRP* C-reactive protein, *TIMP1* Tissue inhibitor of metalloproteinase 1, *TNC* Tenascin C

We examined the association between marker levels and disease severity. For EUVAS-defined disease severity, serum levels of TKT and CD93 tended to be higher as severity increased. Moreover, the levels of both markers were significantly higher in patients with severe disease than in those with localized, early systemic, or generalized disease (Fig. 5a, Additional file 1: Table S11). In terms of RPGN clinical grading, the levels of six markers—TNC, CD93, TKT, TIMP1, CRP, and S100A8/A9—were significantly higher in grades III + IV patients than in grades I + II patients (Fig. 5b, c; Additional file 1: Table S11). TNC, CD93, and TKT showed AUC values of 0.79, 0.76, and 0.74, respectively, for distinguishing between grades I + II and grades III + IV, whereas the AUC values for the other three markers—TIMP1, CRP, and S100A8/A9—were 0.68–0.73. In terms of the FFS 2009, the serum levels of CD93 and TKT were significantly higher in patients with ≥ 3 points than in patients with ≤ 1 or 2 points (Fig. 5d, Additional file 1: Table S11). The levels of the other six markers showed no significant differences between two groups. CD93 and TKT showed AUC values of 0.85 and 0.80, respectively, for discrimination between two groups.

The results of analyses based on these three disease severity classification systems indicated that disease severity in patients with AAV was associated with TKT, CD93, and TNC, which are markers reflecting organ involvement, rather than with markers of inflammation such as CRP and TIMP1.

### Correlations between marker levels

**Fig. 6:**
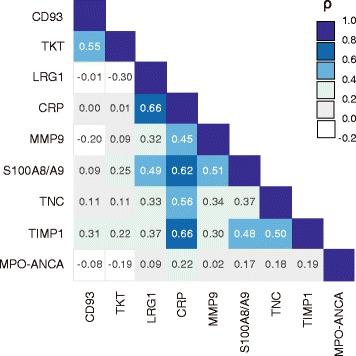
Correlations between marker levels. Correlations between all markers except for myeloperoxidase (MPO)-antineutrophil cytoplasmic antibody (ANCA) were analyzed in the 169 patients with antineutrophil cytoplasmic antibody-associated vasculitis (AAV) before treatment. Correlations between each of the markers and MPO-ANCA were analyzed in the 137 MPO-ANCA-positive patients. Spearman’s rank correlation coefficients for all pairs of biomarkers are shown. Background color indicates strength of association. *CRP* C-reactive protein, *LRG1* Leucine-rich alpha-2-glycoprotein 1, *MMP9* Matrix metalloproteinase 9, *TIMP1* Tissue inhibitor of metalloproteinase 1, *TKT* Transketolase, *TNC* Tenascin C

The correlations among nine markers are summarized in Fig. 6. The levels of five markers—TIMP1, LRG1, S100A8/A9, TNC, and MMP9—correlated well with the CRP value (coefficients 0.45–0.66). In contrast, MPO-ANCA, CD93, and TKT showed no correlation with CRP. None of the markers examined showed any correlation with MPO-ANCA (coefficients ≤ 0.22). A correlation between two markers of renal involvement, TKT and CD93, was recognized (coefficient 0.55). These nine markers were clustered in the following three groups: renal involvement (CD93 and TKT), inflammation (LRG1, CRP, MMP9, S100A8/A9, TNC, and TIMP1), and MPO-ANCA.

### Comparison of marker levels among AAV, RA, SLE, and infectious diseases

**Fig. 7:**
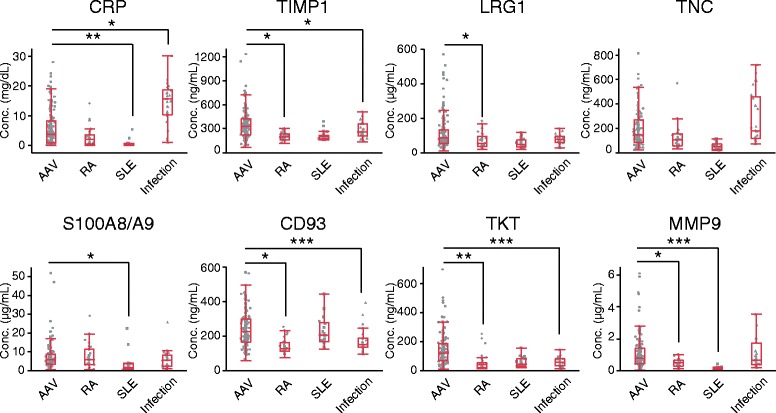
Comparison of marker levels among antineutrophil cytoplasmic antibody-associated vasculitis (AAV), rheumatoid arthritis (RA), systemic lupus erythematosus (SLE), and infectious diseases. The serum levels of each marker were compared among patients with active AAV (*n* = 169), patients with active RA (*n* = 30), patients with active SLE (*n* = 21), and patients with infectious diseases (*n* = 25). The 25 patients with bacterial infections had bacterial pneumonia (*n* = 15), urinary infection (*n* = 4), acute cholecystitis (*n* = 5), and enterocolitis (*n* = 1). Each *dot* represents one patient. Box plots show median and IQR. Whiskers indicate most extreme points within 1.5 times the IQR of the box. *p* Values were determined by analysis of covariance adjusted for age and sex. *p < 0.05; **p < 0.01; ***p < 0.001. *CRP* C-reactive protein, *LRG1* Leucine-rich alpha-2-glycoprotein 1, *MMP9* Matrix metalloproteinase 9, *TIMP1* Tissue inhibitor of metalloproteinase 1, *TKT* Transketolase, *TNC* Tenascin C

We compared the serum levels of the various markers among patients with AAV, RA, SLE, and infectious diseases (Fig. 7). Patients with infectious diseases had significantly higher levels of CRP than patients with active AAV. In contrast, the serum levels of TIMP1, TKT, and CD93 were significantly higher in patients with active AAV than in those with infectious diseases. There were no significant differences in the levels of LRG1, TNC, S100A8/A9, and MMP9 between patients with active AAV and patients with infectious diseases. The circulating level of TKT remained low in patients with RA, SLE, and infectious diseases, although the level in the latter was slightly higher than that in healthy control subjects. These findings suggested that TKT was the best-performing biomarker for discrimination between active AAV and other diseases such as RA, SLE, and infections.

## Discussion

Targeted protein quantification using SRM has emerged as a promising new methodology for clinical biomarker discovery [15–24]. Although reliable SRM assay information on the human serum proteome still has limited public availability, multiple-target SRM quantification without the need for time-consuming antibody development is particularly useful for marker verification. In the present study, we selected 52 proteins as candidate markers for targeted proteomics on the basis of an experimental dataset derived from serum proteomic analysis. Moreover, to maximize the chance of discovering novel marker proteins, 87 vascular endothelium-related proteins were selected as targets on the basis of database search results. A total of 135 proteins were selected as potential marker candidates and subjected to targeted proteomic analysis using SRM. Of these proteins, 74 were successfully quantified. Ultimately, nine proteins—TNC, CRP, TIMP1, LRG1, CD93, S100A8, S100A9, MMP9, and TKT—were identified as candidate biomarkers of disease activity in the SRM assay.

TIMP1 is an endogenous inhibitor of MMPs and an important regulator of extracellular matrix turnover, tissue remodeling, and cellular behavior [41, 42]. TIMP1 is expressed by a variety of cell types in most human tissues. The level of circulating TIMP1 was reportedly elevated in patients with myocardial infarction, sepsis, and various cancers (reviewed in [41–44]). TIMP1 has been also described as elevated in patients with active AAV relative to those in remission or healthy control subjects [9, 45]. Moreover, Monach et al. reported that TIMP1 allows discrimination between mild disease and remission at 6 months, although the AUC was limited at 0.68 [9]. In this study, we showed that TIMP1 was able to distinguish between patients with mildly active AAV and those in remission, whereas CRP and MPO-ANCA were unable to do so. Serum levels of TIMP1 in patients with AAV were significantly higher than those in patients with infectious diseases. In contrast, serum levels of CRP were lower in the former than in the latter. The serum levels of LRG1, MMP9, S100A8/A9, CD93, and TNC were less sensitive than that of TIMP1 for evaluation of disease activity. Our present findings and those of Monach et al. [9] suggest that TIMP1 would be superior to CRP and MPO-ANCA as a biomarker for monitoring the disease activity of AAV. Moreover, because the TIMP1 cutoff point for remission was < 144 ng/ml with a specificity of 94%, this may represent the target level for achieving complete remission.

In the present study, we found two novel markers that reflected renal damage in AAV: TKT and CD93. For prediction of ESRD at 6 months, the cutoff level of TKT was > 229 ng/ml with a sensitivity of 87% and a specificity of 93%, whereas that of CD93 was > 356 ng/ml with a sensitivity of 73% and a specificity of 86%. Moreover, the levels of TKT and CD93 were associated with disease severity as defined by EUVAS, FFS 2009, and RPGN clinical grading. Therefore, high serum levels of TKT and CD93 are able to predict poor overall and ESRD-free survival in patients with AAV. In addition, TKT seems to be a better biomarker than CD93 for reflecting renal involvement because the level of TKT was less elevated than that of CD93 in patients with infectious diseases. TKT is a thiamine diphosphate-dependent enzyme that catalyzes several key reactions in the nonoxidative branch of the pentose phosphate pathway in the cytoplasm [46–48]. TKT is found in all mammalian tissues, and it is highly expressed in cornea, erythrocytes, kidney, and liver [47, 49]. Because TKT is not secreted from cells, its release is a result of cell damage. Therefore, the serum level of TKT in active AAV may be associated with the degree of tissue damage. In addition, the elevated level of circulating TKT in patients with AAV with renal involvement may be due to its high expression in kidney.

CD93 is a type I transmembrane glycoprotein that is upregulated on activated neutrophils, monocytes, and vascular endothelial cells in response to inflammatory mediators such as lipopolysaccharide and tumor necrosis factor-α, and it is shed in soluble form [50, 51]. CD93 is reportedly associated with the risk of coronary artery disease [52, 53]. In the kidney, CD93 is expressed predominantly on infiltrating neutrophils and monocytes, as well as on interstitial and glomerular capillary endothelium [54]. The expression pattern of CD93 can explain the elevation of serum CD93 levels in our study and suggests its involvement in renal inflammation in active AAV.

It has been reported that S100A8/A9 is highly expressed within crescents and in areas of endocapillary proliferation in the kidneys of patients with AAV [55]. However, in the present study, there was no significant difference in the level of circulating S100A8/A9 in patients with or without renal involvement. S100A8/A9 is highly expressed by neutrophils, monocytes, activated macrophages, and microvascular endothelial cells, and it acts as a critical alarmin modulating the inflammatory response after its release from these cells [56]. Therefore, it is suggested that the serum S100A8/A9 level reflects the degree of inflammation more strongly than the degree of renal damage.

The extracellular matrix molecule TNC is highly expressed during embryonic development and tissue repair [57]. TNC expression has been particularly well documented in inflammatory lung conditions such as ILD, bronchial asthma, and tuberculosis [58, 59]. In this study, we found that the serum TNC level reflected the lung infiltration in AAV and was associated with disease severity in terms of the RPGN clinical grading.

Several serum biomarkers that can distinguish between active and inactive AAV and reflect the degree of renal involvement have been reported in large-cohort studies [8–13]. Monach et al. tested 28 markers of inflammation, angiogenesis, and tissue damage and repair in patients enrolled in the RAVE trial, before and 6 months after the start of treatment to distinguish active disease from remission [9]. They identified three promising biomarkers—MMP3, TIMP1, and CXCL13—that best discriminated these two conditions. Pepper et al. showed that the level of S100A8/A9 predicted relapse in patients with PR3-ANCA treated with rituximab in the same trial [13]. Gou et al. reported that plasma levels of C3a, C5a, soluble C5b-9, and Bb were increased in patients with active AAV relative to those in remission [10]. Among serum biomarkers of renal involvement in AAV, the levels of MMP3 and thrombomodulin have been reported to be higher in patients with active renal disease than in those without [8]. Villacorta et al. showed that the baseline serum C3 level had prognostic value for predicting long-term renal and global survival in patients with active renal disease [12]. Brix et al. reported that CCL18 could serve as a biomarker of disease activity and renal relapse in ANCA-associated crescentic glomerulonephritis [11]. Although complement components and several low-content proteins such as cytokines and chemokines (present at the picograms per milliliter level in serum) have been selected as potential candidates in other studies, we eliminated them in the present study through sample pretreatment and the sensitivity limit of the SRM assay. Therefore, it may be possible to evaluate more accurately disease activity and severity, as well as organ damage, in patients with AAV by comparison among the biomarkers identified in the present and previous studies or by creating panels with such biomarkers.

## Conclusions

Targeted proteomics has emerged as a promising new methodology for clinical biomarker discovery. In the present study, we identified promising biomarkers of disease activity and severity, as well as organ involvement, in AAV with a targeted proteomics approach using serum samples collected in a large-cohort Japanese study (RemIT-JAV-RPGN study). Nine proteins—CRP, TIMP1, LRG1, TNC, S100A8/A9, MMP9, CD93, TKT, and MPO-ANCA—were identified as biomarkers of disease activity. Of these, TIMP1 was the best-performing biomarker of disease activity. Moreover, we identified TKT and CD93 as biomarkers for the evaluation of renal involvement and kidney outcome, as well as TNC as a biomarker reflecting lung infiltration in AAV. AAV severity was associated with the levels of TKT, CD93, and TNC (markers reflecting organ involvement) rather than with inflammatory markers. It is expected that these biomarkers will be clinically useful for therapeutic decision-making, monitoring of disease activity, and predicting prognosis.

## Additional files

Additional file 1: Figure S1. Screening of marker candidates in serum samples from patients with AAV. **Figure S2.** Schematic diagram of targeted proteomics using the LC-SRM assay. **Figure S3.** Correlations between the light/heavy ratio of SRM and the ELISA titers of markers. **Figure S4** Correlation of marker levels with total BVAS score of patients with AAV. **Table S1.** Clinical data on healthy donors and patients with RA, SLE, and infectious diseases. **Table S3.** Selected marker candidates for SRM assay development. **Table S4.** A set of established SRM transitions for the detection of marker candidates. **Table S5.** The 20 proteins with a significant change before and at 6 months after treatment in protocol 1 (*n* = 23) and protocol 2 (*n* = 29) in the SRM assay. **Table S6.** Comparison of serum levels of biomarker candidates between the remission group (*n* = 62) and the nonremission group (*n* = 17) at 6 months after treatment. **Table S7.** Marker levels in old healthy donors. **Table S8.** Comparison of serum levels of biomarker candidates between the patients with AAV with (*n* = 120) and without (*n* = 49) renal involvement before treatment. **Table S9.** Comparison of serum levels of biomarker candidates between the patients with AAV with and without lung involvement before treatment. **Table S10.** Comparison of marker levels among MPA, GPA, and EGPA before treatment. **Table S11** Association of marker levels with disease severity in patients with AAV before treatment. (PDF 2221 kb)

Additional file 2: Table S2. Two hundred sixty-seven proteins identified by LC-MS/MS analysis. (XLSX 106 kb)

## Abbreviations

AAV: Antineutrophil cytoplasmic antibody-associated vasculitis
ACR: American College of Rheumatology
ANCA: Antineutrophil cytoplasmic antibody
BVAS: Birmingham Vasculitis Activity Score version 3
CRP: C-reactive protein
ECM: Extracellular matrix
eGFR: Estimated glomerular filtration rate
EGPA: Eosinophilic granulomatosis with polyangiitis
ELISA: Enzyme-linked immunosorbent assay
EMEA: European Medicines Agency
ENT: Ear, nose, and throat
ESRD: End-stage renal disease
EULAR: European League Against Rheumatism
EUVAS: European Vasculitis Study Group
FFS: Five-Factor Score
GFR: Glomerular filtration rate
GPA: Granulomatosis with polyangiitis
ID: Inner diameter
ILD: Interstitial lung disease
L/ES/Ge/Se: Localized/Early systemic/Generalized/Severe
LC-MS/MS: liquid chromatography-tandem mass spectrometry
LR: Likelihood ratio
LRG1: Leucine-rich alpha-2-glycoprotein 1
MMP9: Matrix metalloproteinase 9
MPA: Microscopic polyangiitis
MPO: Myeloperoxidase
MS: Mass spectrometry
MS/MS: Tandem mass spectrometry
n.s.: Not significant
PR3: Proteinase-3
RA: Rheumatoid arthritis
RemIT-JAV-RPGN: Cohort study of remission induction therapy in Japanese patients with antineutrophil cytoplasmic antibody-associated vasculitis and rapidly progressive glomerulonephritis
RPGN: rapidly progressive glomerulonephritis
SLE: Systemic lupus erythematosus
SRM: Selected reaction monitoring
TFA: Trifluoroacetic acid
TIMP1: Tissue inhibitor of metalloproteinase 1
TKT: Transketolase
TNC: Tenascin C
WBC: White blood cell count
