# Supplementary material for: Targeted proteomics reveals promising biomarkers of disease activity and organ involvement in antineutrophil cytoplasmic antibody-associated vasculitis
Source: Arthritis Res Ther. 2017 Sep 29;19:218. doi: 10.1186/s13075-017-1429-3 (PMC5622475; doi:10.1186/s13075-017-1429-3)
Supplement: Supplementary file 1 — Screening of marker candidates in serum samples from patients with AAV. Figure S2. Schematic diagram of targeted proteomics using the LC-SRM assay. Figure S3. Correlations between the light/heavy ratio of SRM and the ELISA titers of markers. Figure S4 Correlation of marker levels with total BVAS score of patients with AAV. Table S1. Clinical data on healthy donors and patients with RA, SLE, and infectious diseases. Table S3. Selected marker candidates for SRM assay development. Table S4. A set of established SRM transitions for the detection of marker candidates. Table S5. The 20 proteins with a significant change before and at 6 months after treatment in protocol 1 (n = 23) and protocol 2 (n = 29) in the SRM assay. Table S6. Comparison of serum levels of biomarker candidates between the remission group (n = 62) and the nonremission group (n = 17) at 6 months after treatment. Table S7. Marker levels in old healthy donors. Table S8. Comparison of serum levels of biomarker candidates between the patients with AAV with (n = 120) and without (n = 49) renal involvement before treatment. Table S9. Comparison of serum levels of biomarker candidates between the patients with AAV with and without lung involvement before treatment. Table S10. Comparison of marker levels among MPA, GPA, and EGPA before treatment. Table S11 Association of marker levels with disease severity in patients with AAV before treatment. (PDF 2221 kb) [file 13075_2017_1429_MOESM1_ESM.pdf]

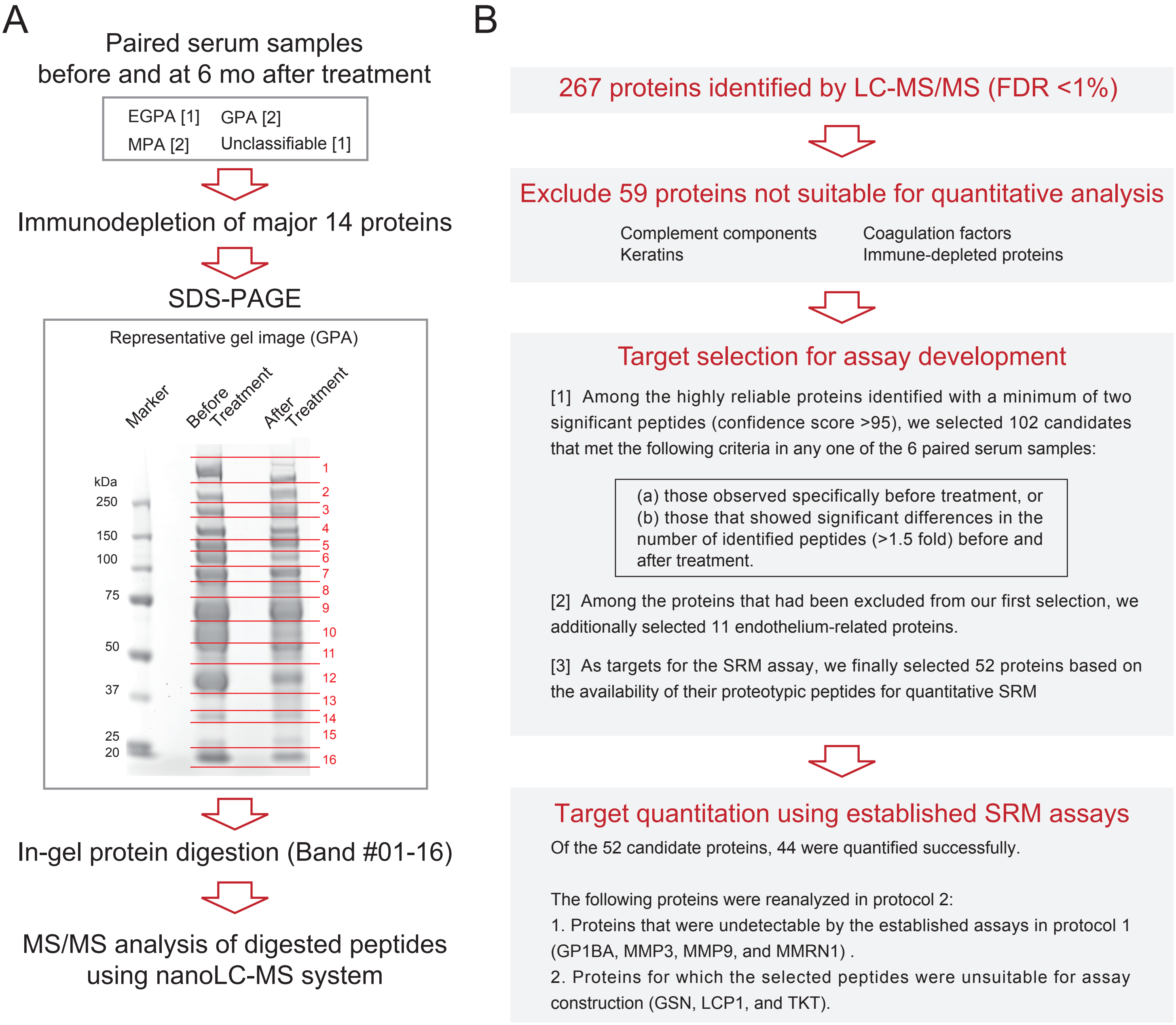

Figure S1. Screening of marker candidates in serum samples from AAV patients. (A) Proteomic profiling of the AAV serum proteome using LC-MS/MS. **(B)** Candidate selection for assay development in protocol 1.

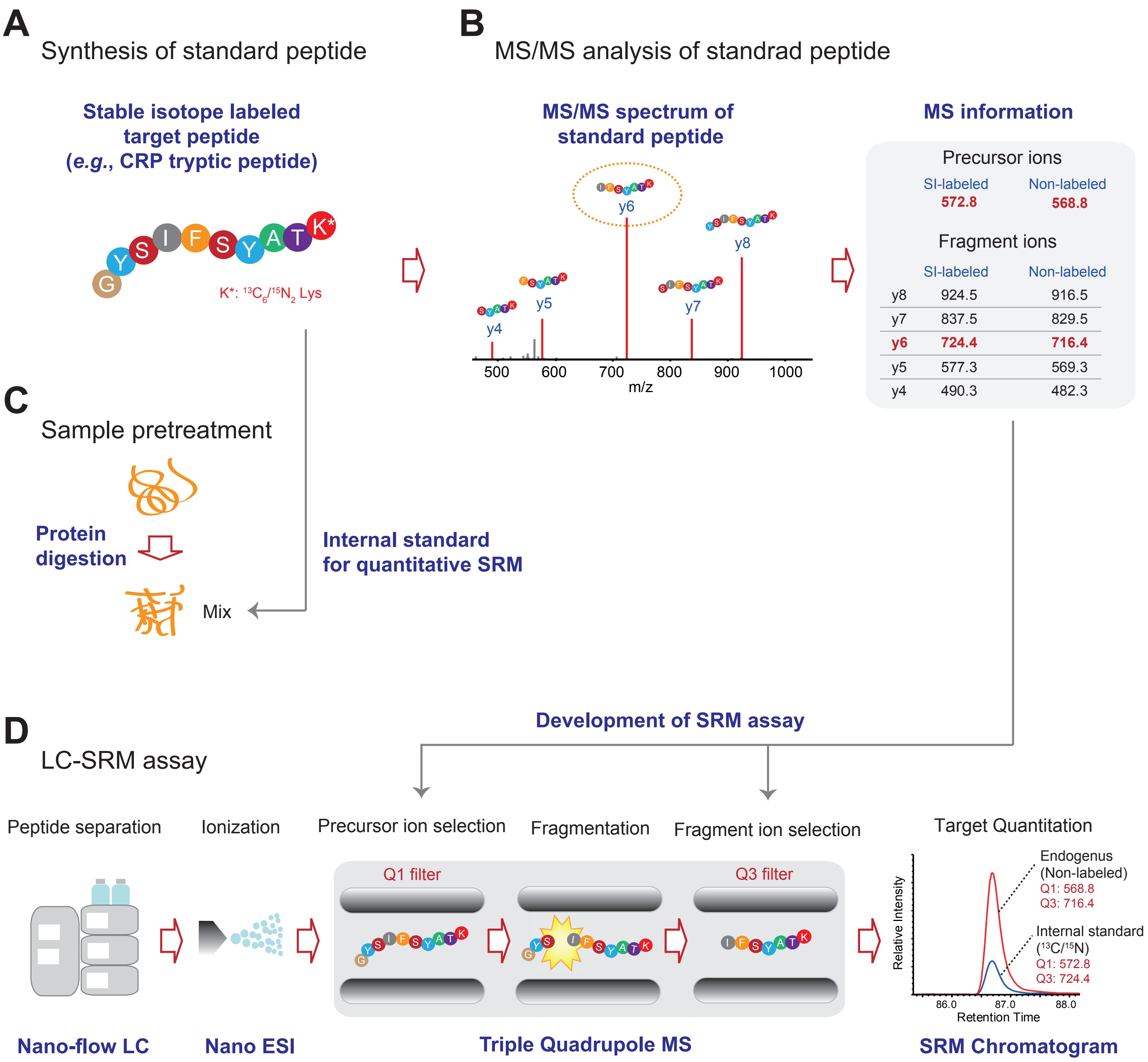

Figure S2. Schematic diagram of targeted proteomics using the LC-SRM assay. (A) Targeted peptides were synthesized with $^{13}C/^{15}N$ labeled amino acids, and used as reference peptides. **(B)** Based on the MS/MS spectra of the SI-labeled reference peptides, we selected a set of fragment ions for each precursor ion and finally established the SRM assays. **(C)** Prior to SRM analysis, serum protein samples were digested with trypsin/Lys-C and mixed with the reference peptide. **(D)** Targeted peptides present in the digested serum sample were detected by LC-SRM analysis. Quantitation of the endogenous target was performed by comparing the area of an endogenous peptide with that of the corresponding SI-labeled reference peptide.

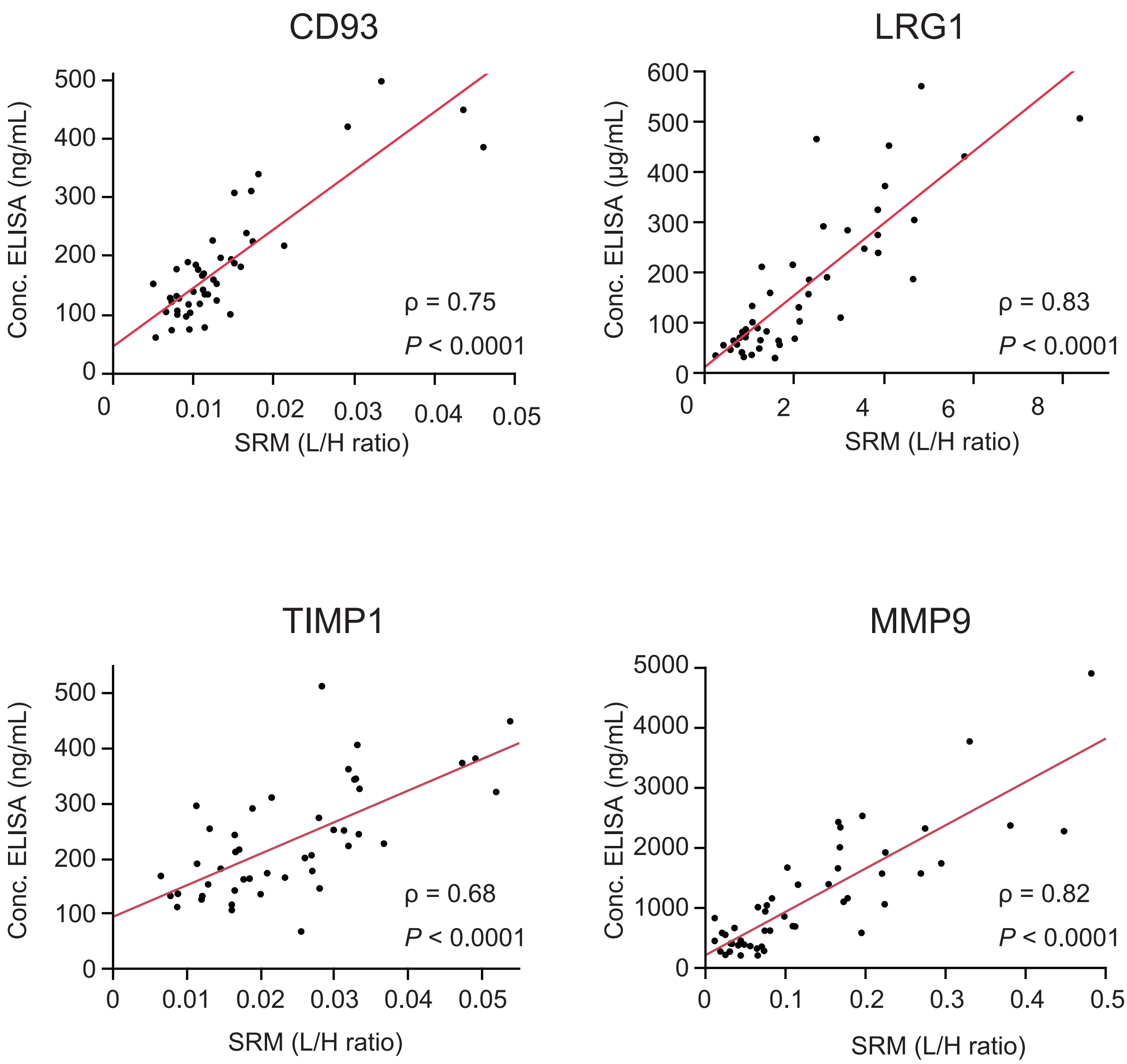

Figure S3. Correlations between the light to heavy ratio of SRM and the ELISA titers of markers. Spearman´s rank correlation coefficients for four representative biomarkers are shown.

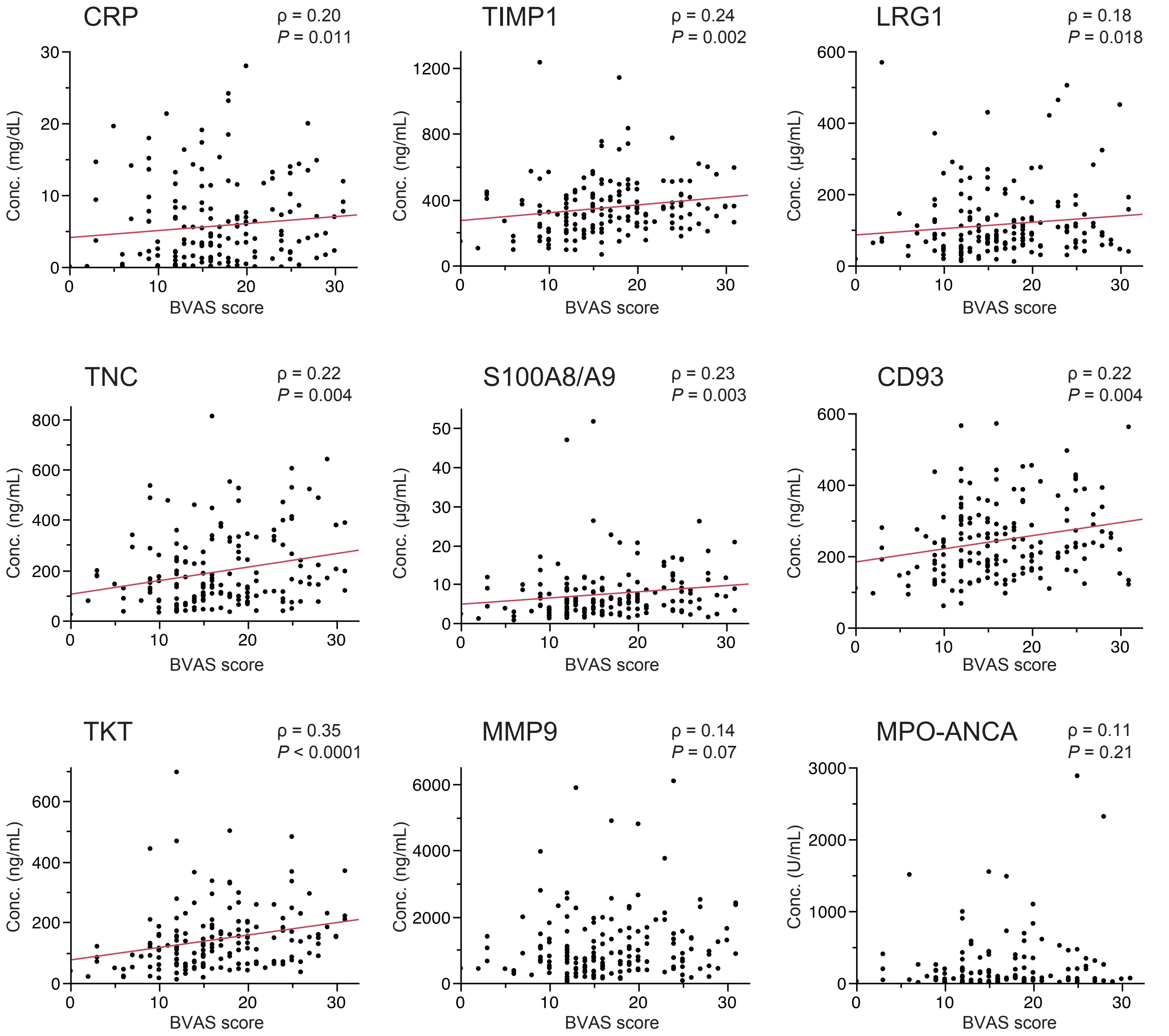

Figure S4. Correlation of marker levels with total BVAS score in AAV patients.
Correlations of marker levels, except for MPO-ANCA with total BVAS score, were analyzed in the 169 AAV patients before treatment. Correlations of MPO-ANCA titers with total BVAS score were analyzed in the 137 MPO-ANCA-positive patients. Spearman´s rank correlation coefficients for all pairs of biomarkers are shown. MPO-ANCA and MMP9 showed no correlation with total BVAS score.

Table S1 Clinical data on healthy donors and patients with RA, SLE, and infectious diseases

Characteristics	Healthy donors	RA	SLE	Infectious diseases
Number	n = 30	n = 30	n = 21	n = 25
Male/female, n/n	9/21	5/25	1/20	16/9
Age, years	29 (25-38)	66 (45-72)	39 (23-45)	79 (74-88)
Disease duration, months		8.5 (3.3-60)	2 (1-4)	
SDAI		16.9 (12.0-30.4)		
SLEDAI score			16 (12-18)	

Values are median (IQR).

RA, rheumatoid arthritis; SLE, systemic lupus erythematosus; SDAI, simplified disease activity index; SLEDAI, systemic lupus erythematosus disease activity index.

Table S2 (See Excel file: Table S2.xlsx)

Table S3. Selected marker candidates for SRM assay development

Protocol 1: Selected proteins based on the following criteria: 1) those specifically observed before treatment, or 2) those that showed significant differences in the number of identified peptides (>1.5 folds) before and after treatment (total 42 proteins)		
Biological function	Symbol	Selected peptides from the peptide dataset observed in our LC-MS/MS analysis
Cell adhesion and extracellular matrix organization	ACTB	GYSFTTTAER
	CDH5	DTGENLETPSSFTIK
	COMP	DTDLDGFPDEK
	FCGBP	LVDPQGPLK
	LRG1	ALGHLDLSGNR
	MMP9 *	FQTFEGDLK
	PRG4	GGSIQQYIYK
	SEPP1	LPTDSELAPR
	TGFBI	IPSETLNR
	THBS1	AQGYSGLSVK
	TNC	ITAQGQYELR
	VASN	YLQGSSVQLR
	VCAM1	SIDGAYTIR
Angiogenesis and wound healing	GP1BA *	GLGELQELYLK
	KNG1	TVGSDTFYSFK
	LPA	GISSTTVTGR
	PFN1	SSFYVNGLTLGGQK
	PLG	LFLEPTR
	PPBP	NIQSLEVIGK
	PROC	LGEYDLR
	PROS1	IETISHEDLQR
	SERPINA10	LFDEINPETK
	SERPINA4	FFSAQTNR
	VWF	TNTGLALR
Inflammatory response	CD5L	LVGGDNLCSGR
	CHI3L1	ILGQQVPYATK
	CRP	GYSIFSYATK
	LBP	ITGFLKPGK
	LTF	DGAGDVAFIR
	LYZ	STDYGIFQINSR
	MASP1	DSDLLSPSDFK
	MBL2	TEGQFVDLTGNR
	PF4	TTSQVRPR
	PROZ	ENFVLTTAK
	S100A8	ALNSIIDVYHK
	SAA2-SAA4	FRPDGLPK
	SERPINA3	ADLSGITGAR
Regulation of metabolism and basic cellular processes	ALDOB	LDQGGAPLAGTNK
	HGFAC	TTDVTQTFGIEK
	MST1	SVTLNQR
	PTGDS	AQGFTEDTIVFLPQTDK
	SHBG	TSSSFEVR
Protocol 1: Endothelium-related proteins (total 10 proteins)		
Biological function	Symbol	Selected peptides from the peptide dataset observed in our LC-MS/MS analysis
Cell adhesion and extracellular matrix organization	CD93	VLAQLLR
	CDH1	MALEVGDYK
	ICAM1	LLGIETPLPK
	LYVE1	DQVETALK
	MMP2	IDAVYEAPQEEK
	MMP3 *	DAVDSAVEK
	MMRN1 *	LVEENALAPDFSK
Angiogenesis and wound healing	MCAM	EETGQVLER
	TIMP1	GFQALGDAADIR
Inflammatory response	B2M	VNHVTLSQPK

Table S3. Continued

	Protocol 2: Endothelium-related proteins selected from public databases† (total 87 proteins)		
Biological function	Symbol	Selected peptides from the proteomics information of public databases§	
Cell adhesion and extracellular matrix organization	AOC3	NLVTMTTAPR	AAALAHLDR
	CD34	LGILDFTEQDVASHQSYSQK	QHVVADTEL
	CDH13	INENTGSVSVTR	DIQGSLQDIFK
	CIB1	AQVPFEQILSLPELK	DGTINLSEFQHVISR
	CLEC14A	GQLPISVTCIADEIGAR	CAVLQATGGVEPAGWK
	COL4A1	AHGQDLGTAGSCLR	ILYHGYSLLYVQGNER
	COL4A2	GLDGYQGPDGPR	SVSIGYLLVK
	CX3CL1	AQDGGPVGTELFR	IPVALLIHYQQNQASCGK
	DLL4	DNLIPAAQLK	AAGSGVFQLQLQEFINER
	EMILIN1	LEQLGGLLANVSGELGGR	APAPASAPPGPSEELLR
	ENG	GPITSAAELNDPQSILLR	TQILEWAAER
	ESM1	CQPSNGEDPFGEEFGICK	TVLDDCGCCR
	FBLN5	YPGAYYIFQIK	DQPFTILYR
	GJA4	LLDQVQEHSTVVGK	ALAAVER
	GP1BB	LLPYLAEDELR	TAHLGANPWR
	ICAM2	ILLDEQAQWK	VPTVEPLDSLTLFLFR
	ICAM3	TFVLPVTPPR	TSLTVVLLR
	ICAM4	GPGWVSYQLLDVR	GSPLAPSGTSVPFWVR
	ITGA4	AYIFSIDEK	SDSAVLLR
	ITGAM	ILVVITDGEK	LFTALFPFEK
	ITGAX	GAVYLFHGVLGPSISPSHSQR	GGQVSVCPLPR
	ITGB2	LGAILTPNDGR	LAENNIQPIFAVTSR
	ITGB7	GEGATQLAPQR	IGFGSFVDK
	JUP	LLNDEDPVVVTK	NEGTATYAAAVLFR
	LAMB2	LQEGQTLEFLVASVPK	GSCYPATGDLLVGR
	LCP1	TENLNDDEK	IGNFSTDIK
	LGALS3	GNDVAFHFNPR	VAVNDAHLLQYNHR
	MADCAM1	GLDTSLGAVQSDTGR	LLPQVSAWAGLR
	MMP3 *	GEDTSMNLVQK	LDSDTLEVMR
	MMP9 *	SLGPALLLLQK	AVIDDAFAR
	MMRN1 *	HSWTIPEDGNSQK	FNPGAESVVLSNSTLK
	PECAM1	STESYFIPEVR	DQNFVILEFPVEEQDR
	PLVAP	VAEEQLVECVK	SLDNLGYNLYHPLGSELASIR
	PODXL	LASVPGSQTVVVK	ATFNPAQDK
	SELE	CAHSPIGEFTYK	FACPEGWTLNGSAAR
	SELP	ALTNEAENWADNEPNNK	STCQFICDEGYSLSGPER
	SULF2	VYHVGLGDAAQPR	SVAIEVDGR
	TPSAB1	EQHLYYQDQLLPVSR	VGIVGGQEAPR
	VASH1	TYFSGNYFR	DGGVPFFVNR
Angiogenesis and wound healing	ANGPT1	QSSLILHGADFSTK	LEIQLLENSLSTYK
	ANPEP	ALEQALEK	DHSAIPVINR
	CD248	LLWIGLQR	VDSLVGAGPASR
	F2R	NESGLTEYR	ATNATLDPR
	FIGF	STNTFFKPPCVNVFR	AASSLEELLR
	FLT4	HATLSLSIPR	DFEQPFINKPDTLLVNR
	GP1BA *	LTSLPLGALR	LTQLNLDR
	KDR	LGPQPLPIHVGELPTPVCK	FLSTLTIDGVTR
	LPL	ITGLDPAGPNFEYAEAPSR	LSPDDADFVDVLHTFTR
	NOS3	AQSYAQQLGR	LSAQAEGLQLLPGLIHVHR
	OLR1	CLSLDAK	GAVSQTYPSGTCAYIQR
	PDGFB	SHSGGELESLAR	TEVFEISR
	PROCR	TLAFPLTIR	CFLGCELPPEGSR
	S1PR1	LTPAQWFLR	TCDILFR
	SELL	SLTEEAENWGDGEPNNK	AEIEYLEK
	SELPLG	SPGLTPEPR	ALGPLLAR
	TFPI	FFFNIFTR	IAYEEIFVK
	THBD	SSVAADVISLLLNGDGGVGR	LAADQHR
	TIE1	VSWSLPLVPGPLVGDGFLLR	FFLTCVSGEAGAGR
Inflammatory response	AIF1L	ANESSPKPVGPPPER	MISEVTGGVSDTISYR
	ANXA1	ALTGHLEEVVLALLK	CATSKPAFFAEK
	AZU1	GPDFFTR	HFCGGALIHAR
	BDKRB1	ANLFISIFLVVAISQDR	SLAPISSSHR
	BDKRB2	LQDWAGSR	LGILSSCQDER
	ELANE	QVFAVQR	VVLGAHNLSR
	PTGIR	NSSLLGLAR	APSAPVGK
	S100A9	LGHPDTLNQGEFK	NIETIINTFHQYSVK

Table S3. Continued

Regulation of metabolism and basic cellular processes	AHSP	DLISAGLK	QELNTLANPFLAK
	ANGPTL1	DNSLELSQLENK	ITGPICVNTK
	ANGPTL2	DHDVYTGNCAHYQK	VFAEYASFR
	CAT	AFYVNVLNEEQR	FNTANDDNVTQVR
	EDN1	ALENLLPTK	SSFHDPK
	EDN2	RPWTEAGAVPSR	TGATTGELLQR
	EDN3	GVSQAPTAAR	ACLHFCTQTLDVSSNSR
	GSN	AGALNSNDAFVLK	TGAQELLR
	HIST1H2AG	VTIAQGGVLPNIQAVLLPK	AGLQFPVGR
	HIST1H3A	STELLIR	YRPGTVALR
	HIST1H4A	DAVTYTEHAK	VFLENVIR
	HTR2B	LTWLTVSTVFQR	KPIQANQYNSR
	KCNA10	GLQILGQTLK	ILISESTNHETAFSK
	KLF2	GLLTPPASPLELLEAKPK	FLLAPPGR
	LSP1	WETGEVQAQSAAK	IDQWLEQYTQAIETAGR
	NCF1	CPHLLDFFK	STATDITGPIILQTYR
	PRSS23	GTPLPTYEEAK	LEVSSSCGPQCHK
	RASD2	TLSSGNCTLSVPAK	FEDQYTPTIEDFHR
	SOD3	AVVVHAGEDDLGR	AGLAASLAGPHSIVGR
	TBXA2R	LSTRPR	SLSLQPQLTQR
	TKT	AVELAANTK	ILATPPQEDAPSVDIANIR

* Proteins analyzed in both protocol 1 and 2 by using different target peptides

† GeneCards (www.genecards.org) and The Human Protein Atlas (www.proteinatlas.org)

§ PeptideAtlas (www.peptideatlas.org) and Plasma Proteome Database (www.plasmaproteomedatabase.org)

Table S4. A set of established SRM transitions for the detection of marker candidates

Protein Name	Peptide Sequence	Light ($^{12}C/^{14}N$-K or R; Endogenous)						Heavy ($^{13}C/^{15}N$-K or R; Internal Standard)					
Protocol 1 (44 proteins)													
Protein Name	Peptide Sequence	Precursor m/z	Precursor Charge	Collision Energy	Product m/z	Product Charge	Fragment Ion	Precursor m/z	Precursor Charge	Collision Energy	Product m/z	Product Charge	Fragment Ion
ACTB	GYSFTTTAER	566.8	2	29.3	678.3	1	y6	571.8	2	29.3	688.4	1	y6
ALDOB	LDQGGAPLAGTNK	621.3	2	31.2	700.4	1	y7	625.3	2	31.2	708.4	1	y7
B2M	VNHVTLSQPK	561.8	2	29.1	673.4	1	y6	565.8	2	29.1	681.4	1	y6
CD93	VLAQLLR	406.8	2	23.5	600.4	1	y5	411.8	2	23.5	610.4	1	y5
CDH1	MALEVGDYK	513.3	2	27.3	823.4	1	y7	517.3	2	27.3	831.4	1	y7
CDH5	DTGENLETPSSFTIK	819.9	2	38.4	880.5	1	y8	823.9	2	38.4	888.5	1	y8
CHI3L1	ILGQQVPYATK	609.3	2	30.8	579.3	1	y5	613.4	2	30.8	587.3	1	y5
COMP	DTDLDGFPDEK	626.3	2	31.4	807.4	1	y7	630.3	2	31.4	815.4	1	y7
CRP	GYSIFSYATK	568.8	2	29.3	716.4	1	y6	572.8	2	29.3	724.4	1	y6
FCGBP	LVDPQGPLK	483.8	2	26.3	754.4	1	y7	487.8	2	26.3	762.4	1	y7
HGFAC	TTDVTQTFGIEK	670.3	2	33.0	694.4	1	y6	674.3	2	33.0	702.4	1	y6
ICAM1	LLGIETPLPK	540.8	2	28.3	854.5	1	y8	544.8	2	28.3	862.5	1	y8
KNG1	TVGSDTFYSFK	626.3	2	31.4	792.4	1	y6	630.3	2	31.4	800.4	1	y6
LBP	ITGFLKPGK	480.8	2	26.2	746.5	1	y7	488.8	2	26.2	762.5	1	y7
LPA	GISSTTVTGR	489.8	2	26.5	721.4	1	y7	494.8	2	26.5	731.4	1	y7
LRG1	ALGHLDLSGNR	576.8	2	29.6	774.4	1	y7	581.8	2	29.6	784.4	1	y7
LTF	DGAGDVAFIR	510.8	2	27.2	777.4	1	y7	515.8	2	27.2	787.4	1	y7
LYVE1	DQVETALK	452.2	2	25.1	660.4	1	y6	456.2	2	25.1	668.4	1	y6
LYZ	STDYGIFQINSR	700.8	2	34.1	764.4	1	y6	705.8	2	34.1	774.4	1	y6
MASP1	DSDLLSPSDFK	612.3	2	30.9	680.3	1	y6	616.3	2	30.9	688.3	1	y6
MBL2	TEGQFVDLTGNR	668.8	2	32.9	675.3	1	y6	673.8	2	32.9	685.4	1	y6
MCAM	EETGQVLER	530.8	2	28.0	802.4	1	y7	535.8	2	28.0	812.5	1	y7
MMP2	IDAVYEAPQEEK	696.3	2	33.9	830.4	1	y7	700.3	2	33.9	838.4	1	y7
MST1	SVTLNQR	409.2	2	23.6	631.4	1	y5	414.2	2	23.6	641.4	1	y5
PFN1	SSFYVNGLTLGGQK	735.9	2	35.3	887.5	1	y9	739.9	2	35.3	895.5	1	y9
PLG	LFLEPTR	438.3	2	24.6	615.3	1	y5	443.3	2	24.6	625.4	1	y5
PPBP	NIQSLEVIGK	550.8	2	28.7	873.5	1	y8	554.8	2	28.7	881.5	1	y8
PRG4	GGSIQQYIYK	578.8	2	29.7	842.4	1	y6	582.8	2	29.7	850.5	1	y6
PROC	LGEYDLR	433.2	2	24.5	566.3	1	y4	438.2	2	24.5	576.3	1	y4
PROS1	IETISHEDLQR	447.6	3	21.9	549.8	2	y9	450.9	3	21.9	554.8	2	y9
PROZ	ENFVLTTAK	511.8	2	27.3	533.3	1	y5	515.8	2	27.3	541.3	1	y5
S100A8	ALNSIIDVYHK	636.9	2	31.8	774.4	1	y6	640.9	2	31.8	782.4	1	y6
SAA2-SAA4	FRPDGLPK	465.3	2	25.6	686.4	1	b6	474.3	2	25.6	696.4	1	b6
SEPP1	LPTDSELAPR	549.8	2	28.6	493.3	2	y9	554.8	2	28.6	498.3	2	y9
SERPINA10	LFDEINPETK	603.3	2	30.6	945.5	1	y8	607.3	2	30.6	953.5	1	y8
SERPINA4	FFSAQTNR	485.7	2	26.3	676.3	1	y6	490.7	2	26.3	686.3	1	y6
SHBG	TSSSFEVR	456.7	2	25.3	724.4	1	y6	461.7	2	25.3	734.4	1	y6
TGFBI	IPSETLNR	465.3	2	25.6	719.4	1	y6	470.3	2	25.6	729.4	1	y6
THBS1	AQGYSGLSVK	505.3	2	27.0	810.4	1	y8	509.3	2	27.0	818.4	1	y8
TIMP1	GFQALGDAADIR	617.3	2	31.1	717.4	1	y7	622.3	2	31.1	727.4	1	y7
TNC	ITAQGQYELR	589.8	2	30.1	765.4	1	y6	594.8	2	30.1	775.4	1	y6
VASN	YLQGSSVQLR	575.8	2	29.6	746.4	1	y7	580.8	2	29.6	756.4	1	y7
VCAM1	SIDGAYTIR	498.3	2	26.8	795.4	1	y7	503.3	2	26.8	805.4	1	y7
VWF	TNTGLALR	423.2	2	24.1	630.4	1	y6	428.2	2	24.1	640.4	1	y6
Protocol 2 (30 proteins)													
Protein Name	Peptide Sequence	Light ($^{12}C/^{14}N$-K or R; Endogenous)						Heavy ($^{13}C/^{15}N$-K or R; Internal Standard)					
		Precursor m/z	Precursor Charge	Collision Energy	Product m/z	Product Charge	Fragment Ion	Precursor m/z	Precursor Charge	Collision Energy	Product m/z	Product Charge	Fragment Ion
ANPEP *	ALEQALEK	451.3	2	25.1	717.4	1	y6	455.3	2	25.1	725.4	1	y6
ANXA1	ALTGHLEEVVLALLK	536.0	3	26.7	711.4	2	y13	538.7	3	26.7	715.4	2	y13
AOC3 *	NLVTMTTAPR	552.3	2	28.7	876.5	1	y8	557.3	2	28.7	886.5	1	y8
CAT	AFYVNVLNEEQR	741.4	2	35.5	788.4	1	y6	746.4	2	35.5	798.4	1	y6
CD248 *	LLWIGLQR	499.8	2	26.9	772.4	1	y6	504.8	2	26.9	782.5	1	y6
CDH13 *	INENTGSVSVTR	638.8	2	31.9	705.4	1	y7	643.8	2	31.9	715.4	1	y7
ELANE	QVFAVQR	424.2	2	24.1	620.4	1	y5	429.2	2	24.1	630.4	1	y5
FLT4	HATLSLSIPR	547.8	2	28.6	886.5	1	y8	552.8	2	28.6	896.5	1	y8
GP1BA *	LTSLPLGALR	520.8	2	27.6	626.4	1	y6	525.8	2	27.6	636.4	1	y6
GSN *	AGALNSNDAFVLK	660.4	2	32.6	893.5	1	y8	664.4	2	32.6	901.5	1	y8
HIST1H4A	DAVTYTEHAK	567.8	2	29.3	849.4	1	y7	571.8	2	29.3	857.4	1	y7
ICAM2	ILLDEQAQWK	622.3	2	31.3	904.4	1	y7	626.3	2	31.3	912.4	1	y7
ICAM3	TFVLPVTPPR	563.8	2	29.2	878.5	1	y8	568.8	2	29.2	888.6	1	y8
JUP *	LLNDEDPVVVTK	671.4	2	33	446.3	1	y4	675.4	2	33	454.3	1	y4
LAMB2	LQEGQTLEFLVASVPK	880.0	2	40.5	600.4	1	y6	884.0	2	40.5	608.4	1	y6
LCP1 *	TENLNDDEK	539.2	2	28.3	847.4	1	y7	543.2	2	28.3	855.4	1	y7
LPL	ITGLDPAGPNFEYAEAPSR	669.0	3	34	559.3	1	y5	672.3	3	34	569.3	1	y5
LSP1	WETGEVQAQSAAK	468.9	3	23.1	575.3	1	y6	471.6	3	23.1	583.3	1	y6
MMP3	GEDTSMNLVQK	611.3	2	30.9	819.4	1	y7	615.3	2	30.9	827.5	1	y7
MMP9 *	SLGPALLLLQK	576.9	2	29.6	952.6	1	y9	580.9	2	29.6	960.6	1	y9
MMRN1	HSWTIPEDGNSQK	500.2	3	24.8	437.7	2	y8	502.9	3	24.8	441.7	2	y8
PODXL *	LASVPGSQTVVVK	642.9	2	32	914.5	1	y9	646.9	2	32	922.5	1	y9
PROCR *	TLAFPLTIR	516.3	2	27.4	817.5	1	y7	521.3	2	27.4	827.5	1	y7
S100A9 *	LGHPDTLNQGEFK	485.9	3	24	722.3	1	y6	488.6	3	24	730.4	1	y6
SELL *	SLTEEAENWGDGEPNNK	945.4	2	42.9	472.3	1	y4	949.4	2	42.9	480.3	1	y4
SELP	ALTNEAENWADNEPNNK	965.4	2	43.6	472.3	1	y4	969.4	2	43.6	480.3	1	y4
SOD3	AVVVHAGEDDLGR	669.3	2	33	832.4	1	y8	674.3	2	33	842.4	1	y8
SULF2	VYHVGLGDAAQPR	691.9	2	33.8	884.5	1	y9	696.9	2	33.8	894.5	1	y9
TFPI *	FFFNIFTR	546.3	2	28.5	797.4	1	y6	551.3	2	28.5	807.4	1	y6
TKT *	AVELAANTK	458.8	2	25.4	746.4	1	y7	462.8	2	25.4	754.4	1	y7

* Proteins quantified in paired serum samples from ≥20 patients

Table S5　The 20 proteins with a significant change before and at 6 months after treatment in protocol 1 (n = 23) and protocol 2 (n = 29) in the SRM assay

Marker	Highly active (L/H ratio)	Remission (L/H ratio)	*P* value
Protocol 1			
Down-regulated proteins			
CD93	0.015 (0.013-0.017)	0.01 (0.008-0.011)	<0.0001*
CRP	6.8 (0.9-10.6)	0.15 (0.03-0.29)	<0.0001*
LRG1	3.1 (1.7-3.9)	1.3 (0.9-1.7)	<0.0001*
S100A8	0.28 (0.12-0.44)	0.1 (0.08-0.14)	0.0003*
TIMP1	0.032 (0.025-0.045)	0.018 (0.013-0.026)	<0.0001*
TNC	0.037 (0.028-0.045)	0.018 (0.014-0.024)	<0.0001*
Up-regulated proteins			
HGFAC	0.12 (0.1-0.15)	0.18 (0.14-0.25)	0.0006*
MCAM	0.011 (0.009-0.016)	0.022 (0.02-0.027)	<0.0001*
PROC	0.09 (0.07-0.12)	0.15 (0.11-0.19)	<0.0001*
SEPP1	0.14 (0.12-0.21)	0.21 (0.15-0.32)	0.0008*
SERPINA4	0.19 (0.1-0.24)	0.36 (0.25-0.44)	<0.0001*
Protocol 2			
Down-regulated proteins			
MMP9	0.026 (0.013-0.044)	0.011 (0.007-0.02)	0.001†
S100A9	0.15 (0.1-0.26)	0.07 (0.04-0.1)	<0.0001†
TKT	0.22 (0.15-0.35)	0.12 (0.1-0.2)	<0.0001†
Up-regulated proteins			
ANPEP	0.71 (0.56-0.86)	1.09 (0.68-1.31)	0.0011†
CDH13	0.032 (0.024-0.038)	0.049 (0.037-0.053)	0.0002†
GSN	0.67 (0.41-1.04)	1.53 (0.97-1.87)	<0.0001†
JUP	0.92 (0.61-1.28)	1.67 (1.07-2.08)	0.0002†
PODXL	0.006 (0.004-0.006)	0.007 (0.006-0.01)	<0.0001†
TFPI	0.018 (0.014-0.027)	0.029 (0.02-0.036)	0.0074†

Values are median (IQR). *P* values were determined by Wilcoxon signed-rank test. Statistically significance was determined by **P*<0.05/44 (protocol 1) or †*P*<0.05/15 (protocol 2) by Bonferroni correction.

L/H ratio; light to heavy ratio, HGFAC, hepatocyte growth factor activator; MCAM, melanoma cell adhesion molecule; PROC, protein C; SEPP1, selenoprotein P; SERPINA4, serpin family A member 4; ANPEP, aminopeptidase N; CDH13, cadherin 13; GSN, gelsolin; JUP, junction plakoglobin; PODXL, podocalyxin like; TFPI, tissue factor pathway inhibitor.

Table S6 Comparison of serum levels of biomarker candidates between the remission group (n = 62) and the non-remission group (n = 17) at 6 months after treatment

Marker	Remission	Non-remission	*P* value
CRP (mg/dL)	0.058 (0.011-0.163)	0.027 (0.013-0.111)	0.99
TIMP1 (ng/mL)	156 (134-175)	180 (147-196)	0.02*
LRG1 (μg/mL)	40.1 (29.8-62.0)	38.5 (27.4-56.1)	0.70
TNC (ng/mL)	47.0 (37.0-68.3)	58.1 (25.1-93.1)	0.58
S100A8/A9 (μg/mL)	2.2 (1.2-3.8)	2.9 (1.9-3.6)	0.23
CD93 (ng/mL)	124 (103-164)	169 (108-243)	0.09
TKT (ng/mL)	52.0 (28.9-91.5)	89.3 (58.9-137)	0.01*
MMP9 (ng/mL)	566 (330-839)	720 (574-963)	0.09
MPO-ANCA (U/mL)※	0.8 (0.48-2.2)	1.5 (0.38-3.3)	0.52

Values are median (IQR).

*$P<0.05$ by analysis of covariance adjusted for age, sex, and four distinct AAV groups (MPA, GPA, EGPA, and unclassifiable disease).

※Only MPO-ANCA positive patients; n = 60 (remission group, n = 48; non-remission group, n = 12)

Table S7 Marker levels in old healthy donors

	Old healthy donors (n=16)	Healthy donors (n=30)	*P* value
Age, years	72 (67-79)	29 (25-38)	
TIMP1 (ng/mL)	147 (129-159)	132 (115-148)	0.1
CD93 (ng/mL)	142 (110-158)	125 (103-139)	0.17
TKT (ng/mL)	23.8 (13.8-39.9)	33.1 (15.1-42.6)	0.51

Values are median (IQR).

P values are determined by Mann-Whitney U test.

Old healthy donors: Sex (male, n=12; female, n=4); median eGFR 67 (IQR: 60-71) mL/min/1.73m^2.

Table S8 Comparison of serum levels of biomarker candidates between the AAV patients with (n = 120) and without renal involvement (n = 49) before treatment

Marker	Renal involvement		*P* value
	+	-	
CRP (mg/dL)	3.7 (0.9-7.8)	4.3 (1.1-9.3)	0.24
TIMP1 (ng/mL)	344 (246-437)	316 (174-403)	0.61
LRG1 (μg/mL)	86.3 (53.0-132)	84.5 (59.6-145)	0.92
TNC (ng/mL)	145 (81.6-277)	145 (95.2-234)	0.90
S100A8/A9 (μg/mL)	5.7 (3.6-8.9)	6.3 (3.0-8.6)	0.88
CD93 (ng/mL)	255 (199-340)	163 (126-193)	0.0001*
TKT (ng/mL)	147 (81.6-277)	88.2 (52.0-131)	0.0006*
MMP9 (ng/mL)	758 (427-1324)	907 (445-1557)	0.40
MPO-ANCA (U/mL)※	71.7 (30.2-335)	80.1 (43.5-188)	0.74

Values are median (IQR).

**P*<0.05 by analysis of covariance adjusted for age, sex, and four distinct AAV groups (MPA, GPA, EGPA, and unclassifiable disease).

※Only MPO-ANCA positive patients; n = 137 (with renal involvement, n = 106; without renal involvement, n = 31).

Table S9 Comparison of serum levels of biomarker candidates between the AAV patients with and without lung involvement before treatment

Marker	No lung involvement	Lung infiltration		Nodules or cavities	
	n = 95	n = 25	*P* value†	n = 11	*P* value§
CRP (mg/dL)	3.2 (0.3-7.7)	4.3 (1.2-9.1)	0.19	6.9 (4.5-8.1)	0.93
TIMP1 (ng/mL)	308 (213-396)	372 (267-457)	0.06	360 (341-416)	0.09
LRG1 (μg/mL)	86.1 (52.1-134)	88.0 (50.2-129)	0.76	153 (88.5-208)	0.98
TNC (ng/mL)	132 (75.3-186)	197 (111-301)	0.03*	305 (157-346)	0.22
S100A8/A9 (μg/mL)	5.3 (3.2-8.6)	6.3 (3.2-8.6)	0.40	7.8 (5.4-9.4)	0.29
CD93 (ng/mL)	212 (164-297)	231 (169-352)	0.26	205 (152-236)	0.25
TKT (ng/mL)	115 (62.5-172)	137 (78.7-203)	0.16	76.6 (63.5-111)	0.58
MMP9 (ng/mL)	780 (429-1388)	746 (362-1099)	0.42	1554 (668-1916)	0.39
MPO-ANCA (U/mL)※	63.2 (26.5-202)	80.7 (46.8-294)	0.41	139 (97.7-437)	0.31

Values are median (IQR).

*$P<0.05$ by analysis of covariance adjusted for age, sex, and four distinct AAV groups (MPA, GPA, EGPA, and unclassifiable disease). †No lung involvement vs lung infiltration. §No lung involvement vs nodules or cavities.

※Only MPO-ANCA positive patients: no lung involvement, n = 77, lung infiltration, n = 23; nodules or cavities, n = 6.

Table S10 Comparison of marker levels among MPA, GPA, and EGPA before treatment

Marker	MPA (n = 105)	GPA (n = 36)	EGPA (n = 25)
CRP (mg/dL)	3.63 (0.62-8.93)	5.45 (1.98-8.34)	2.13 (0.41-6.32)
TIMP1 (ng/mL)	350 (227-465)	314 (253-365)	299 (153-395)
LRG1 (μg/mL)*	83.2 (48.2-120)	103 (71.4-186)	79.9 (53.3-127)
TNC (ng/mL)*	142 (75.1-242)	209 (126-335)	131 (87.3-197)
S100A8/A9 (μg/mL)	5.6 (3.6-8.6)	6.8 (3.5-10.6)	5.5 (2.8-8.5)
MMP9 (ng/mL)*	728 (394-1191)	1142 (675-1909)	746 (428-1557)
CD93 (ng/mL)*, †	263 (203-352)	187 (126-231)	160 (130-193)
TKT (ng/mL)*, §	143 (85-191)	83 (57.3-136)	115 (68.9-149)

Values are median (IQR).

P values were determined by analysis of covariance adjusted for age, sex. Statistically significance was determined by <0.05/3 by Bonferroni correction: *MPA vs GPA. †MPA vs EGPA. §GPA vs EGPA.

Table S11 Association of marker levels with disease severity in AAV before treatment

Marker	EUVAS			RPGN			FFS		
	L / ES / Ge n = 138	Severe n = 31	*P* value	I + II n = 126	III + IV n = 43	*P* value	≤1 + 2 n = 124	3≤ n = 45	*P* value
CRP (mg/dL)	4.0 (0.6-8.8)	3.6 (2.1-6.6)	0.09	2.9 (0.4-7.2)	6.7 (3.5-13.8)	0.002*	4.6 (1.2-9.2)	2.4 (0.3-5.6)	0.005*
TIMP1 (ng/mL)	333 (225-424)	350 (244-448)	0.43	305 (212-398)	405 (331-515)	0.001*	343 (224-425)	312 (227-434)	0.05
LRG1 (μg/mL)	86.6 (53.8-141)	75.3 (54.1-109)	0.45	83.7 (50.6-133)	96.7 (70.9-136)	0.14	90.0 (60.7-146)	70.1 (46.7-108)	0.17
TNC (ng/mL)	144 (83.0-248)	152 (90.6-368)	0.20	129 (75.2-184)	301 (193-430)	<0.0001*	145 (86.3-237)	157 (75.9-332)	0.41
S100A8/A9 (μg/mL)	5.8 (3.3-8.9)	5.6 (4.3-8.7)	0.32	5.4 (3.1-8.3)	8.0 (5.3-12.0)	0.003*	6.0 (3.6-8.9)	5.3 (3.2-8.1)	0.20
CD93 (ng/mL)	211 (162-281)	292 (209-390)	0.006*	200 (150-270)	293 (233-388)	0.0001*	197 (151-252)	337 (279-405)	<0.0001*
TKT (ng/mL)	110 (63.5-161)	184 (144-296)	0.0002*	109 (62.0-155)	183 (120-276)	0.0005*	95.5 (61.6-153)	184 (140-294)	<0.0001*
MMP9 (ng/mL)	847 (441-1474)	625 (285-1107)	0.02*	758 (434-1371)	884 (367-1598)	0.92	909 (453-1557)	534 (285-908)	0.002*
MPO-ANCA (U/mL)※	73.7 (31.7-212)	88.6 (38.9-327)	0.26	80.4 (32.4-211)	69.8 (40.9-311)	0.76	80.4 (34.2-252)	67.2 (31.4-277)	0.83

Values are median (IQR).

**P*<0.05 by analysis of covariance adjusted for age, sex, and four distinct AAV groups (MPA, GPA, EGPA, and unclassifiable disease).

EUVAS, European Vasculitis Study Group, L/ES/Ge, Localized/Early systemic/Generalized; RPGN, rapidly progressive glomerulonephritis; FFS, Five-Factor Score.

※Only MPO-ANCA positive patients: EUVAS (L/ES/Ge, n = 110; Severe, n = 27); RPGN (I + II, n = 96; III + IV, n = 41); FFS (≤1 + 2, n = 94; 3≤, n = 43)
